# Transcriptome reveals the roles and potential mechanisms of lncRNAs in the regulation of albendazole resistance in *Haemonchus contortus*

**DOI:** 10.1186/s12864-024-10096-6

**Published:** 2024-02-17

**Authors:** Xindi Chen, Tengyu Wang, Wenrui Guo, Xu Yan, Huilin Kou, Yu Yu, Chunxia Liu, Wa Gao, Wenlong Wang, Rui Wang

**Affiliations:** 1 Key Laboratory of Animal Disease Clinical Diagnosis and Treatment Technology, College of Veterinary Medicine, Inner Mongolia Agricultural University, Ordos Street, Hohhot, 010018 Inner Mongolia Municipality China; 2 Key Laboratory of Animal Disease Clinical Diagnosis and Treatment Technology, College of Life Science, Inner Mongolia Agricultural University, Hohhot, 010018 Inner Mongolia Municipality China; 3 Inner Mongolia Key Laboratory of Tick-Borne Zoonotic Infectious Disease, Department of Medicine, Hetao College, Bayan Nur, 015000 Inner Mongolia Autonomous Region China

**Keywords:** *Haemonchus contortus*, Albendazole, Drug resistance, lncRNA, ceRNA

## Abstract

**Background:**

*Haemonchus contortus* (*H. contortus)* is the most common parasitic nematode in ruminants and is prevalent worldwide. *H. contortus* resistance to albendazole (ABZ) hinders the efficacy of anthelmintic drugs, but little is known about the molecular mechanisms that regulate this of drug resistance. Recent research has demonstrated that long noncoding RNAs (lncRNAs) can exert significant influence as pivotal regulators of the emergence of drug resistance.

**Results:**

In this study, transcriptome sequencing was conducted on both albendazole-sensitive (ABZ-sensitive) and albendazole-resistant (ABZ-resistant) *H. contortus* strains, with three biological replicates for each group. The analysis of lncRNA in the transcriptomic data revealed that there were 276 differentially expressed lncRNA (DElncRNA) between strains with ABZ-sensitive and ABZ-resistant according to the criteria of |log2Foldchange|≥ 1 and FDR < 0.05. Notably, MSTRG.12969.2 and MSTRG.9827.1 exhibited the most significant upregulation and downregulation, respectively, in the resistant strains. The potential roles of the DElncRNAs included catalytic activity, stimulus response, regulation of drug metabolism, and modulation of the immune response. Moreover, we investigated the interactions between DElncRNAs and other RNAs, specifically MSTRG.12741.1, MSTRG.11848.1, MSTRG.5895.1, and MSTRG.14070.1, involved in regulating drug stimulation through *cis*/*trans*/antisense/lncRNA‒miRNA–mRNA interaction networks. This regulation leads to a decrease (or increase) in the expression of relevant genes, consequently enhancing the resistance of *H. contortus* to albendazole. Furthermore, through comprehensive analysis of competitive endogenous RNAs (ceRNAs) involved in drug resistance-related pathways, such as the mTOR signalling pathway and ABC transporter signalling pathway, the relevance of the MSTRG.2499.1-novel-m0062-3p-*HCON_00099610* interaction was identified to mainly involve the regulation of catalytic activity, metabolism, ubiquitination and transcriptional regulation of gene promoters. Additionally, quantitative real-time polymerase chain reaction (qRT–PCR) validation indicated that the transcription profiles of six DElncRNAs and six DEmRNAs were consistent with those obtained by RNA-seq.

**Conclusions:**

The results of the present study allowed us to better understand the changes in the lncRNA expression profile of ABZ-resistant *H. contortus*. In total, these results suggest that the lncRNAs MSTRG.963.1, MSTRG.12741.1, MSTRG.11848.1 and MSTRG.2499.1 play important roles in the development of ABZ resistance and can serve as promising biomarkers for further study.

**Supplementary Information:**

The online version contains supplementary material available at 10.1186/s12864-024-10096-6.

## Background

*Haemonchus contortus* is a common parasitic gastrointestinal nematode of small ruminants that can cause host anaemia and chronic clinical symptoms, seriously impair animal health and production performance, and cause economic losses in the animal breeding industry. Its prevention and treatment depend mainly on the use of chemical drugs such as albendazole, ivermectin and levamisole [1]. With the unreasonable use of many deworming drugs, drug resistance to a variety of deworming drugs has emerged worldwide (Pyrénées, Cuba, Guadeloupe, US Virgin Islands, Zhaosu) [2–6], making the prevention and control of parasitic diseases more difficult.

The resistance of *H. contortus* to albendazole and other anthelmintic drugs was first reported in 1986 [7]. Related early research conducted on the complete genome of *H. contortus* revealed that the development of albendazole resistance is closely related to β-tubulin. Different studies have reported that single nucleotide polymorphisms (SNPs) occurring at codons 167, 198, and 200 of the isotype-1 *β-tubulin* gene are associated to the development of parasite drug resistances [8–13]. Furthermore, the development of drug resistance has also been associated with P-glycoprotein-encoding genes (*P-gps*) in nematodes, which facilitate the expulsion of drugs to the extracellular space, leading to drug resistance [14]. Nevertheless, by analogy with other eukaryotes, drug resistance in nematodes could result from a range of different mechanisms in addition to functional mutations in specific genes [15]. Numerous studies have substantiated the utility of noncoding RNAs (ncRNAs) as diagnostic markers for various ailments and these noncoding transcripts appear to play important roles in the resistance to targeted therapeutic drugs in various diseases.

The wide range of applications using high-throughput sequencing technology provides new opportunities for exploring drug resistance. Long noncoding RNAs (lncRNAs) are RNAs that are longer than 200 nt and have little protein-coding ability; these molecules can affect messenger RNA (mRNA) [16, 17] splicing and maturation, trafficking or localization, and stability and may also be involved in dosage compensation, genomic imprinting, epigenetic modifications and gene expression regulation [18–25]. Moreover, some lncRNAs have been shown to play important roles in the regulation of drug resistance in bacteria and chemotherapy-related resistance in human cancers [26, 27]. In recent years, lncRNAs have been highly studied in the field of life science, and research on lncRNAs in model organisms such as humans and mice has increased in depth [28–31]. There are few reports on lncRNAs related to parasites [32–34]; in particular, the role of lncRNAs in the generation of drug resistance in parasites has not been explored, and the underlying functional mechanism involved remains to be discovered.

Our research team has long been engaged in the study of common anthelmintic resistance in *H. contortus*. Zhao [35] provided an initial exploration of the expression of lncRNAs in ABZ-resistant strains. However, due to the relatively low quality of the *H. contortus* genome data at that time, only an incomplete draft genome, potentially lacking a large number of valuable regulatory genes, was available. At the same time, the samples of strains used in the previous sequencing were obtained as natural isolates in the field. To obtain better experimental and analytical results, standard sensitive strains with clearer backgrounds and international recognition were selected for this study. Through comparison with the complete genome map of *H. contortus*, a more in-depth and multiangle analysis was carried out.

To explore transcriptome differences and identify albendazole-related lncRNAs, we compared the lncRNA expression levels of ABZ-sensitive and ABZ-resistant *H. contortus* adults, and predicted DElncRNA–mRNA interactions. In addition, competitive endogenous RNA (ceRNA) regulatory network analysis was carried out to infer the functions of the differentially expressed lncRNAs (DElncRNAs). Such ceRNA network-based research related to drug resistance pathways offers a structured approach for comprehensively analysing complex datasets and facilitating a holistic understanding of multiple interacting groups. Our results suggest that lncRNAs may play key roles in conferring drug resistance to *H. contortus* and provide a reference for revealing the molecular mechanism related to drug resistance in *H. contortus* and related drug development.

## Results

### RNA quality testing and transcriptome sequencing

**Fig. 1:**
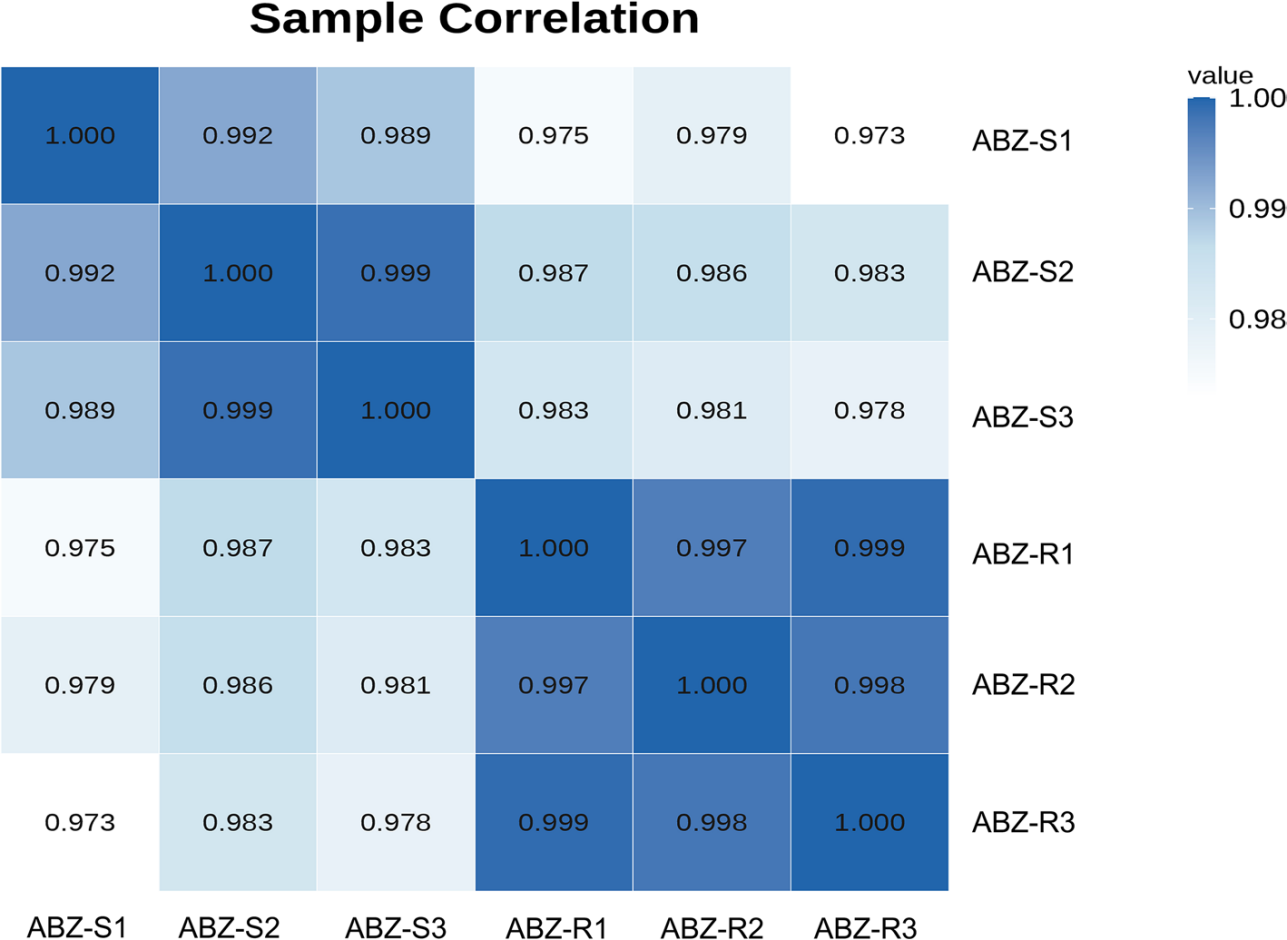
Heatmap of lncRNA relationships of the ABZ-sensitive and ABZ-resistant strains of *H. contortus*. ABZ-SX: ABZ-sensitive strain X; ABZ-RX: ABZ-resistant strain X

All the RNA samples were subjected to quality testing with NanoDrop 2000 and Agilent 4200 instruments and gel electrophoresis (Additional file 1: Figure S1). RNA sequencing produced 11.39 Gb and 11.34 Gb of raw data were obtained for the ABZ-sensitive and ABZ-resistant strains, respectively, comprising 11,403,175,500, 11,219,175,600, 11,560,346,400, 11,497,460,700, 11,372,158,800 and 11,151,021,000 paired-end raw reads from the six samples. The obtained raw sequencing data were subjected to quality control. Bases with an average quality Q20 accounted for 95.1% and 95.1% of the total base obtained from the six libraries (three replicates for each sample). The proportion of bases with an average quality of Q30 reached 88.4% and 88.3%, respectively, and all the RNA samples were of high quality (RIN > 9, OD260/280 = 1.8 ~ 2.2, 28S:18S ≥ 1) (Additional file 1: Table S2). The lncRNA Pearson correlation coefficient between ABZ-sensitive and ABZ-resistant strains showed high consistency of biological replicates in the same group and good separation between sensitive and resistant strains (Fig. 1). The above results indicate that the sequencing data are of good quality and meet the needs for subsequent analyses.

### Classification and detection of lncRNAs

**Fig. 2:**
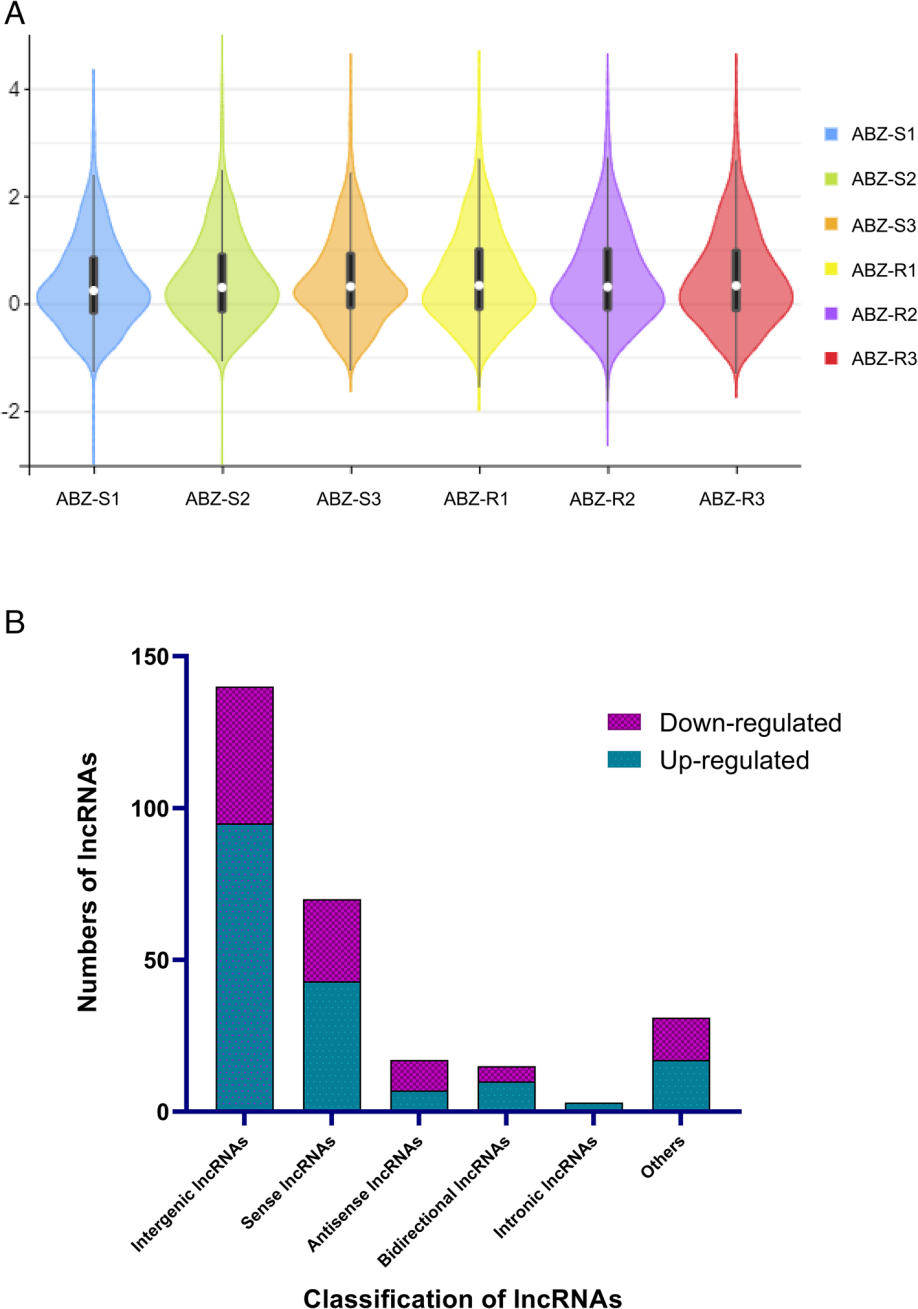
LncRNA comparative analysis. **A** Visualization of lncRNA abundance differences between ABZ-sensitive and ABZ-resistant strains. **B** Distribution of lncRNA types. ABZ-SX: ABZ-sensitive strain X; ABZ-RX: ABZ-resistant strain X

The expression abundance of lncRNAs in different samples was determined based on the expression level determined by FPKM and count statistics (Fig. 2A). One of the mechanisms of action of lncRNAs is through interaction with neighbouring coding genes; therefore, classification of putative lncRNAs according to related protein-coding genes may provide a preliminary indication of potential lncRNA function. Our data revealed that among the 276 DElncRNAs in the ABZ-sensitive and ABZ-resistant comparisons, intergenic lncRNAs and sense lncRNAs exhibited predominant expression, with 140 and 70 instances, respectively. Conversely, intronic lncRNAs account for a mere 3 occurrences (Fig. 2B).

### LncRNA expression profiles of ABZ-sensitive and ABZ-resistant strains

**Fig. 3:**
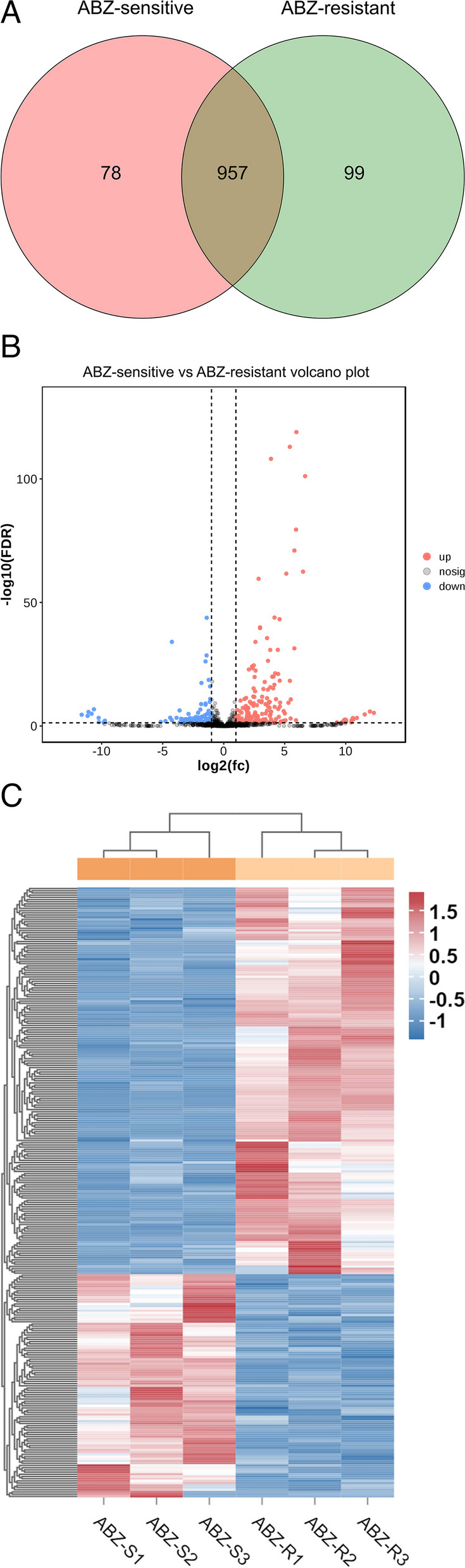
DElncRNAs analysis between ABZ-sensitive and ABZ-resistant strains of *H. contortus*. **A** Venn diagram showing the common and unique DElncRNA transcripts between ABZ-sensitive and ABZ-resistant strains. **B** Volcano plot of DElncRNAs between ABZ-sensitive and ABZ-resistant strains. Red, blue and grey dots represent upregulated, downregulated, and not significantly different lncRNAs, respectively. **C** Clustering of the DElncRNAs between ABZ-sensitive and ABZ-resistant strains. The upregulated and downregulated genes are coloured red and blue, respectively. ABZ-SX: ABZ-sensitive strain X; ABZ-RX: ABZ-resistant strain X

A total of 1,227 novel lncRNAs without any preexisting annotation information were identified in the ABZ-sensitive and ABZ-resistant *H. contortus* populations. Based on the expression levels of these lncRNAs in the six samples, a Venn diagram was constructed to determine the intersection of the expression levels within each group (an lncRNA with a count value greater than 0 in two or more samples was considered to be expressed in that group). A total of 78 lncRNAs were specifically expressed in ABZ-sensitive strains, and 99 lncRNAs were specifically expressed in ABZ-resistant strains. The lncRNAs for which the false discovery rate (FDR) was less than 0.05 and the absolute fold change was ≥ 1 were considered differentially expressed. A total of 276 DElncRNAs were identified. Among them, 175 DElncRNAs were upregulated and 101 DElncRNAs were downregulated between the ABZ-sensitive and ABZ-resistant strains. The top three most upregulated were MSTRG.12969.2, MSTRG.9782.1 and MSTRG.11528.1, and the top three most downregulated were MSTRG.9827.1, MSTRG.963.1 and MSTRG.7719.1, respectively (Additional file 1: Table S3). Among them, all the lncRNAs were considered new transcripts, and 59.7% of the lncRNAs were less than 500 nt in length. A volcano plot was generated to evaluate the expression levels of lncRNAs in ABZ-sensitive and ABZ-resistant strains, and a heatmap was generated to compare the expression patterns of the lncRNAs in the two groups. Moreover, through the FPKM value and count value statistics, the abundance of lncRNAs in different samples was determined based on the expression level (Fig. 3A-C). The FPKM values of 67.8% and 77.2% of the lncRNAs were greater than or equal to 1, respectively, indicating that lncRNAs were highly expressed and increased during the process of *H. contortus* drug resistance development, and the expression of MSTRG.11920.3 was the highest of all the identified lncRNAs.

### Functional annotation of DElncRNA colocated genes

**Fig. 4:**
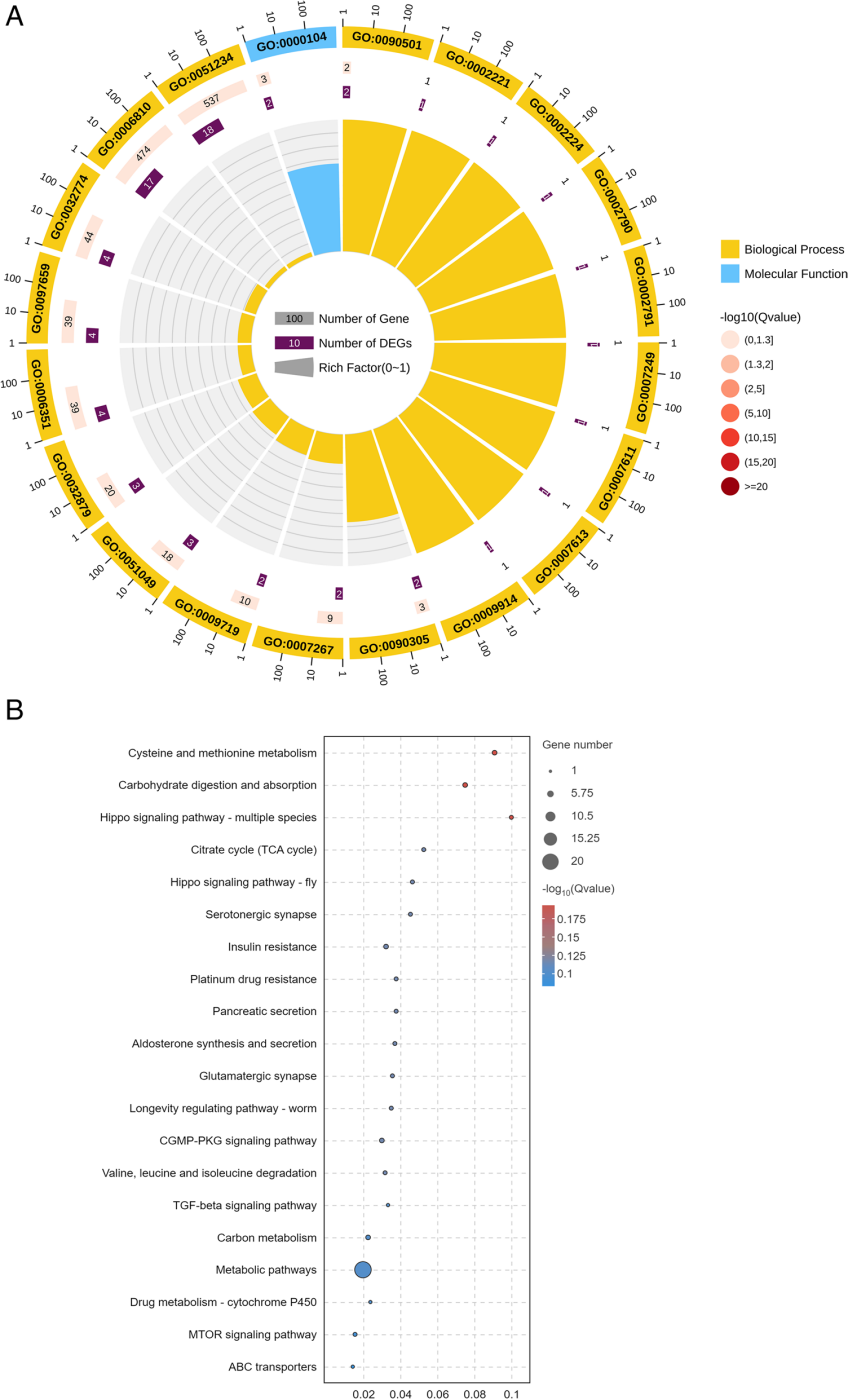
Enrichment analysis of DElncRNAs (cis-regulatory, top 20). **A** GO functional enrichment of DElncRNAs. **B** KEGG (www.kegg.jp/kegg/kegg1.html) functional enrichment of the DElncRNAs

Using a genome colocation method, lncRNAs can be functionally annotated by analysing the functions of their adjacent protein-coding genes (10 kb upstream and downstream). Out of the pool of 276 DElncRNAs, a subset of 72 lncRNAs were identified as *cis*-regulatory elements, and a total of 85 interactions with coding genes were detected (61 positive correlations and 24 negative correlations). The results of the GO database annotation of the *cis*-regulatory target genes of the lncRNAs showed that 76 GO terms were significantly enriched, including 65 biological process, 3 cellular component and 8 molecular function terms. Notably, several terms related to drug resistance were identified: protein kinase C activity (GO:0004697), regulation of transmembrane transporter activity (GO:0022898) and regulation of insulin secretion (GO:0050796). Moreover, MSTRG.12742 and MSTRG.12741.1 were identified in this analysis. KEGG [36] analysis revealed that 17 DElncRNA target genes were significantly enriched in metabolic pathways. In addition, the target genes corresponding to MSTRG.9919.1, MSTRG.8672.2, MSTRG.10520.1 and MSTRG.11545.1 were also significantly enriched in drug resistance-related pathways, namely, drug metabolism-cytochrome P450, ABC transporter and mTOR signalling (Fig. 4A, B).

### Functional annotation of DElncRNA coexpressed genes

**Fig. 5:**
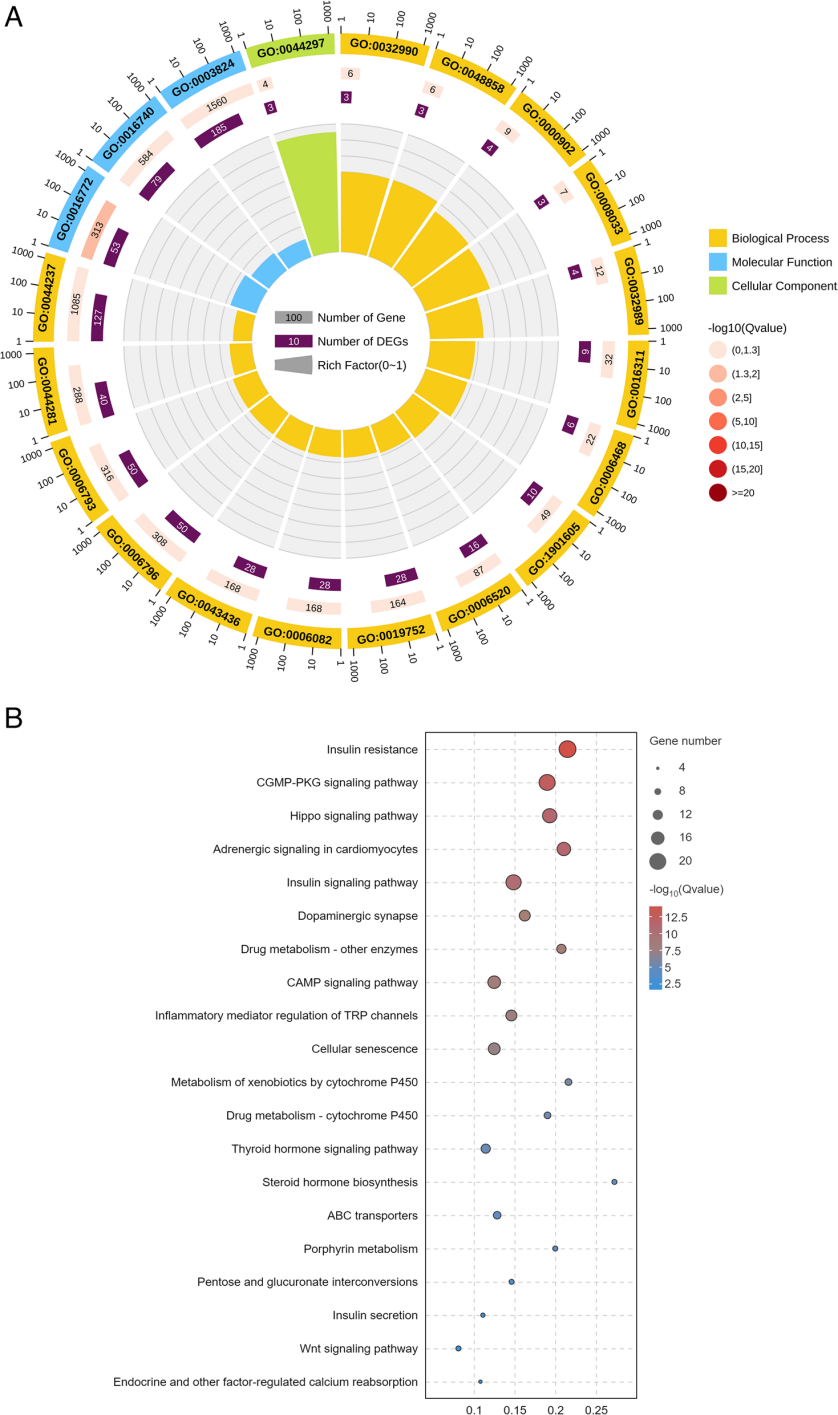
Enrichment analysis of the DElncRNAs (trans-regulatory, top 20). **A** GO functional enrichment of DElncRNAs. **B** KEGG functional enrichment of DElncRNAs

By predicting the correlation between the expression of DElncRNAs and protein-coding genes, the protein-coding genes coexpressed with DElncRNAs were analysed, and the functional annotation of the lncRNAs was carried out (Fig. 5A, B). A total of 263 lncRNAs were identified to form 40,410 interactions with 2,143 coding genes. The lncRNA MSTRG.11848.1 had the strongest correlation with *trans*-regulatory target genes, and its target genes *HCON_00141050* (*CYP6B2*) and *HCON_00073880* (*CYP2c70*) were related to drug metabolism. In addition, *HCON_00110560* (*ABCC1*), which was positively correlated with 25 DElncRNAs and negatively correlated with 23 DElncRNAs (Additional file 1: Table S4), was enriched in the typical resistance-related pathway ABC transporters.

### Functional annotation of DElncRNA–mRNA complementary genes

**Fig. 6:**
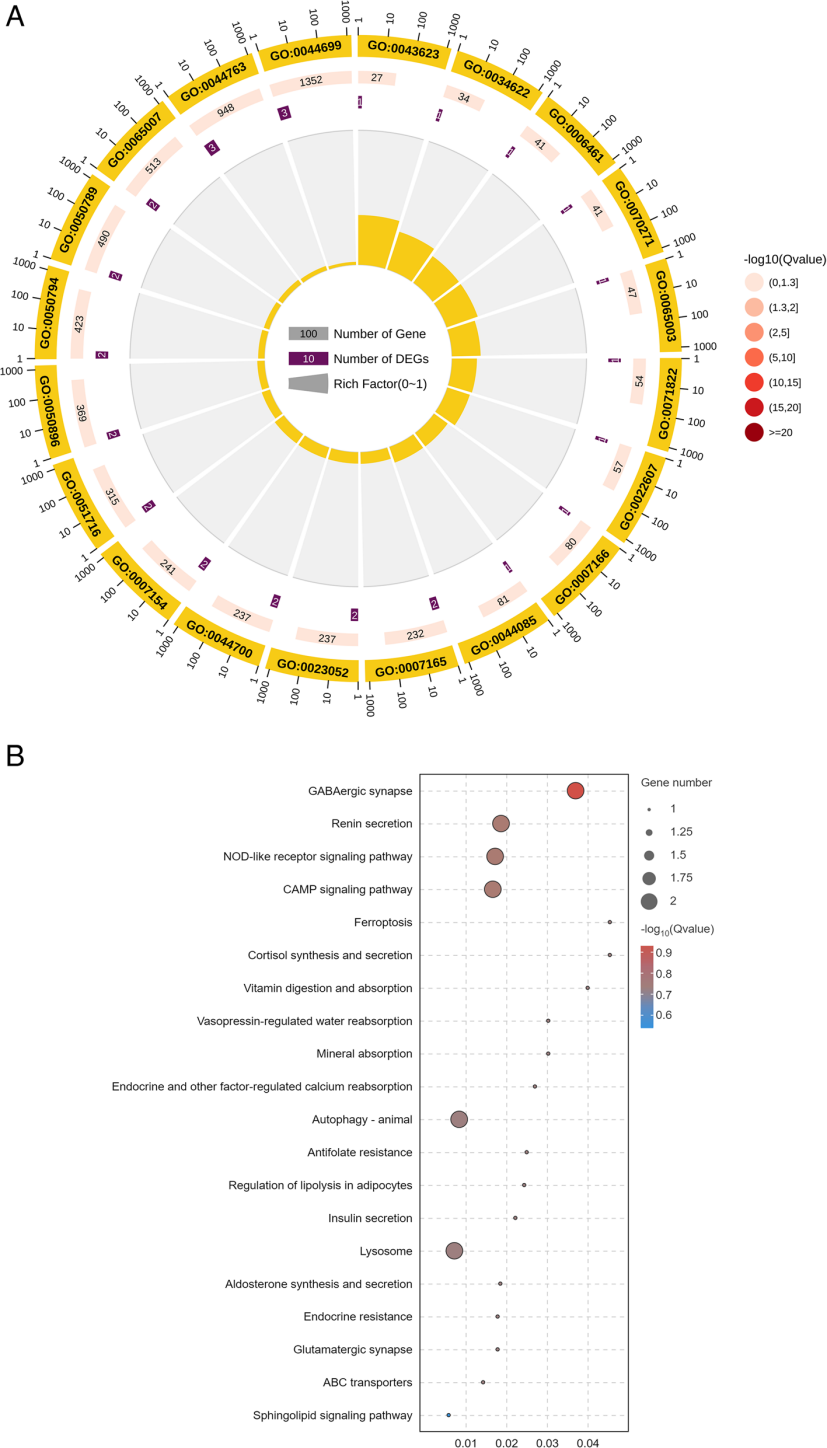
Enrichment analysis of the DEGs (antisense prediction, top 20). **A** GO functional enrichment of DElncRNAs. **B** KEGG functional enrichment of DElncRNAs

Among the ABZ-sensitive and ABZ-resistant strains, 154 lncRNAs and 146 mRNAs were found to form 169 complementary pairs, of which 19 DElncRNAs and 19 differentially expressed genes (DEGs) formed 19 targeting pairs. The Gene Ontology functional annotations of mRNAs complementary to lncRNAs were mainly focused on biological regulation (GO:0065007), response to stimuli (GO:0050896) and catalytic activity (GO:0003824) (Fig. 6A). MSTRG.5895.1 is located in the antisense genomic region of the DEG *HCON_00068400*, indicating that it has a potential functional regulatory relationship with translation-related processes. KEGG pathway analysis revealed that the target genes of the DElncRNAs are involved in 69 pathways, and the five pathways with the most significant P values were morphine addiction, GABAergic synapses, renin secretion, NOD-like receptor signalling and ovarian ferroptosis (Fig. 6B).

### Response of the lncRNA‒miRNA‒mRNA regulatory network to albendazole

**Fig. 7:**
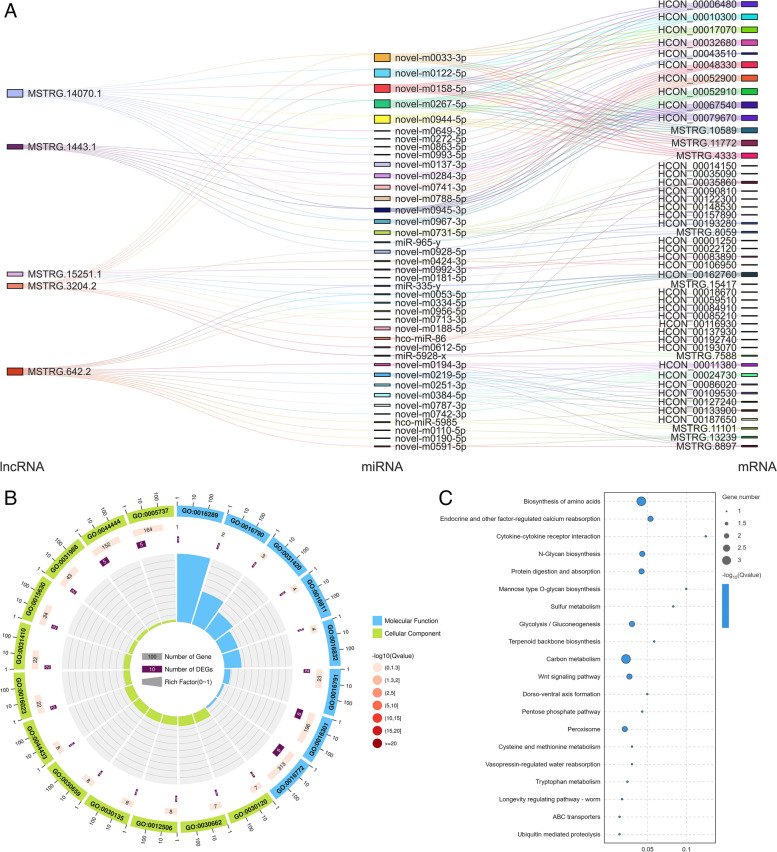
Association analysis of lncRNA‒miRNA‒mRNA interactions. **A** Sankey diagram of the lncRNA connectivity (top 5). **B** GO functional enrichment of DElncRNAs associated with miRNA‒mRNAs (top 20). **C** KEGG functional enrichment of DElncRNA‒associated miRNA‒mRNAs (top 20)

Based on the ceRNA model, Cytoscape software was used to visualize the results of the mutual targeting regulatory network of all the significantly DElncRNAs, miRNAs and mRNAs. The network included 219 (152 upregulated and 67 downregulated) DElncRNAs targeted 186 miRNAs, which in turn bound to 280 mRNAs. A small number of lncRNAs (14%) can bind only a single miRNA, and some miRNAs were targeted by multiple (2–19) lncRNAs. Among them, MSTRG.14070.1 binds the largest number of miRNAs. There were 19, 16, 11, 10 and 9 regulatory connections to the top five nodes (MSTRG.14070.1, MSTRG.642.2, MSTRG.15251.1, MSTRG.3204.2 and MSTRG.1443.1) (Fig. 7A). These factors can prevent the negative regulatory effect of novel-m0033-3p on collagen synthesis in the stratum corneum, the inhibition of carbohydrate transmembrane transporter activity by novel-m0219-5p, and the negative regulatory effect of novel-m0992-3p on prolonging the lifespan of *H. contortus*. According to the GO and KEGG analyses (Fig. 7B, C), a total of 235 entries and 106 signalling pathways were annotated. LncRNA MSTRG.12264.1, which exhibited strong connectivity, were enriched mainly in the insulin signalling pathway and were related to drug resistance.

### CeRNA network analysis of pathways related to drug resistance

**Fig. 8:**
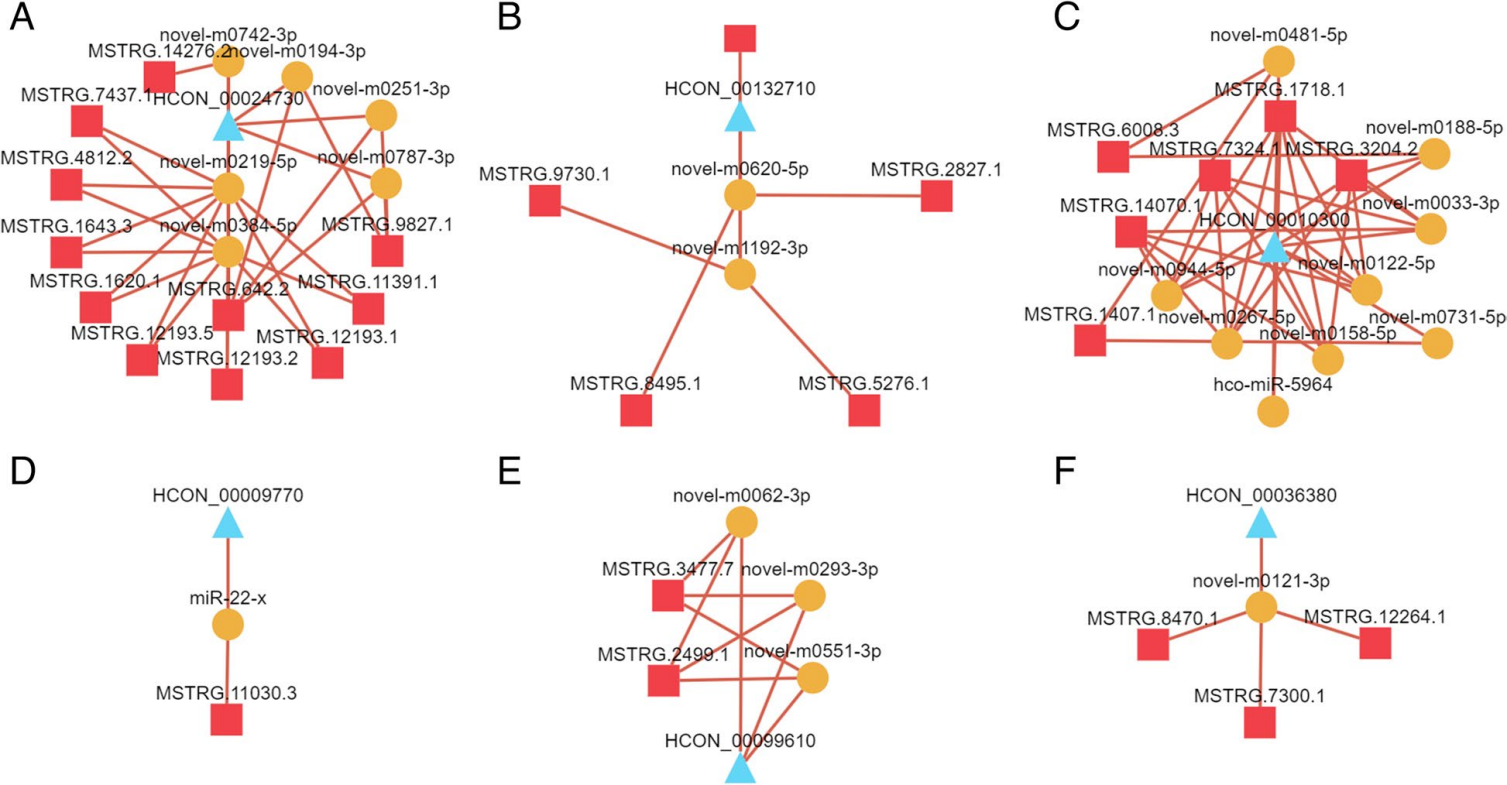
lncRNA‒miRNA–mRNA regulatory network of drug resistance-related signalling pathways. **A** FoxO signalling pathway. **B** MAPK signalling pathway. **C** The PI3K–Akt signalling pathway/NF-κB signalling pathway. **D** mTOR signalling pathway. **E** ABC transporter pathway. **F** Insulin signalling pathway. The blue rectangles, yellow circles and blue triangles represent lncRNAs, miRNAs and mRNAs, respectively

The reported drug resistance-related pathways, the connectivity analysis (Fig. 8A-F) of DElncRNAs, DEmRNAs, and DEmiRNAs in ceRNA relationships, and KEGG enrichment results showed that FoxO, MAPK, PI3K–Akt/NF-κB, mTOR, ABC transporters and insulin signalling pathways involved DEGs *HCON_00024730* (*FBXO32*), *HCON_00132710* (*bs*), *HCON_00010300* (*SYK*), *HCON_00009770* (*FZD4*), *HCON_00099610* (*ABCD2*) and *HCON_00036380* (*ptp-3*), which were involved in 11, 5, 6, 1, 2 and 3 regulatory networks, respectively. *HCON_00024730*, *HCON_00132710* and *HCON_00010300* had strong connectivity in the lncRNA‒miRNA–mRNA networks, which further indicated that they had stronger potential regulatory ability. In addition, the ABC transporter, an efflux transporter, is recognized as a drug resistance gene that is closely related to resistance to ivermectin [37] and albendazole [38]. In brief, the DEmRNAs targeted by DElncRNAs were highly unique, the network demonstrated the multi-target effects of miRNAs, and the key regulatory factors in ceRNA networks were further identified.

### Validation of lncRNA and mRNA expression

**Fig. 9:**
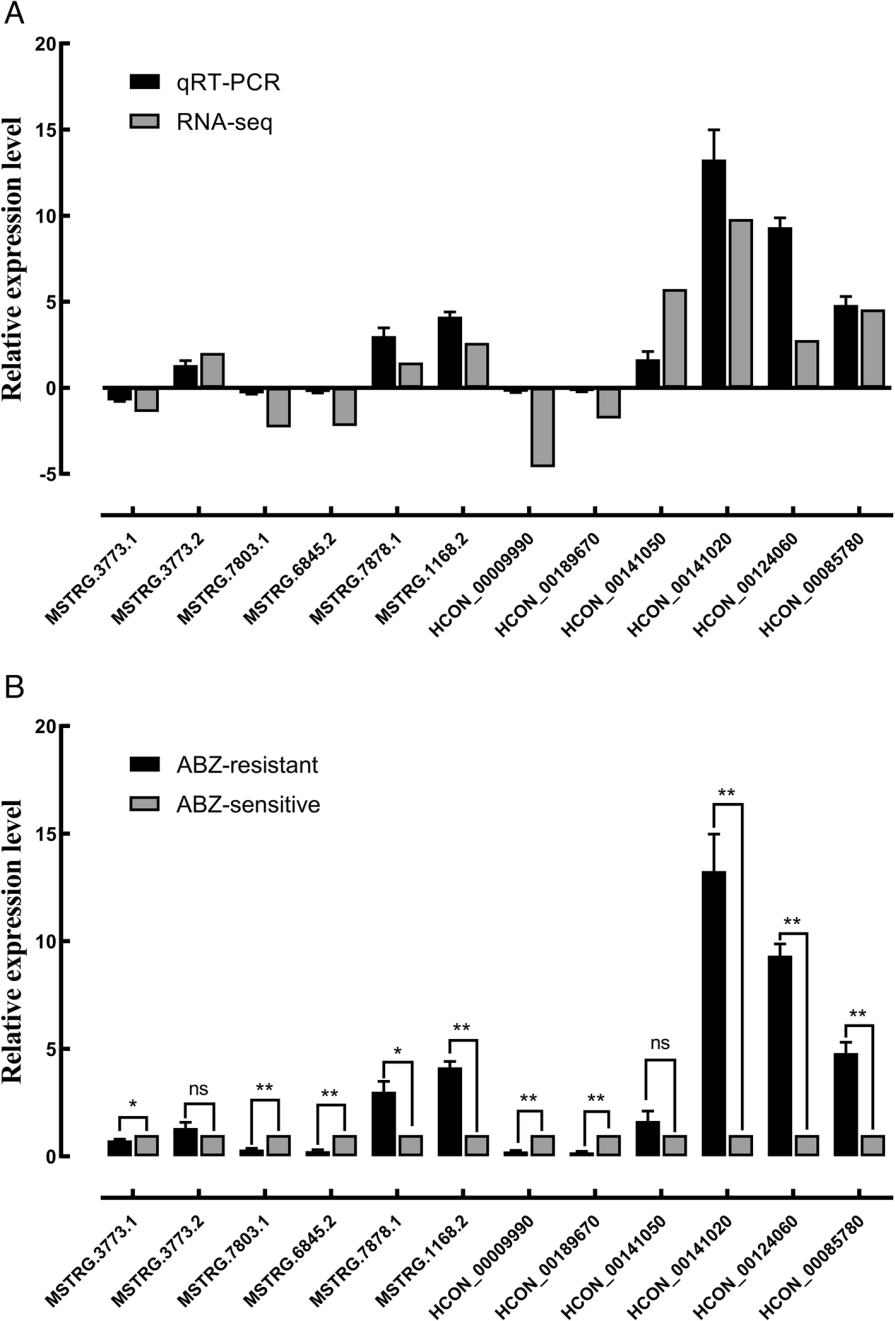
Validation of RNA-seq data using qRT‒PCR. **A**. RNA-seq, **B**. qRT‒PCR. qRT‒PCR validation of DElncRNAs and DEmRNAs in adult ABZ-sensitive and ABZ-resistant strains. Note: *: *P* < 0.05; **: *P* < 0.01; ns: not significant

Six upregulated (downregulated) DElncRNAs/DEmRNAs were randomly selected from the comparison group of ABZ-sensitive and ABZ-resistant strains for qRT-PCR to verify the expression trend. The changes in the expression of the six DElncRNAs and six DEmRNAs in the comparison group were confirmed by qRT‒PCR, and the trend was consistent with the transcriptome results. This validation further confirmed the stability and reliability of the transcriptome sequencing results (Fig. 9A, B).

## Discussion

Albendazole resistance is common in *H. contortus*. LncRNAs have a variety of biological functions and are also closely associated with the emergence of drug resistance [39, 40]. By analysing the functions of lncRNAs, we can better understand the complex biological processes and regulatory mechanisms involved in drug resistance. In previous work, our team analysed ABZ-sensitive and ABZ-resistant strains isolated from Ulanqab, Inner Mongolia Autonomous Region. A total of 6,505 novel lncRNAs were identified, and 168 DElncRNAs were obtained (92 upregulated and 76 downregulated). The target genes of the DElncRNAs were annotated to drug- resistance-related pathways, specifically, drug metabolism-other enzymes, drug metabolism-cytochrome P450 and metabolism of xenobiotics by cytochrome P450 [35]. In the present study, the known lncRNAs were not predicted or compared with the reference genome and database. According to position, length and coding ability, 1227 novel lncRNA transcripts were identified in the genome. Compared with those in the sensitive strains, the transcriptional profiles of the lncRNAs in the ABZ-resistant strains significantly altered. We identified 276 DElncRNAs, of which 175 were increased in expression, while the remaining DElncRNAs were significantly decreased in expression. This phenomenon suggests that under stress from albendazole exposure, most lncRNAs that promote the development of drug resistance act through upregulation of expression. Considering the results of the present work, comparing with Zhao’s results [35], the total number of identified novel lncRNAs here decreased, and the number of DElncRNAs increased. The reason may be that the replacement of incomplete reference genomes in this study resulted in the filtering out of several low-quality, irrelevant genes, while additional valuable genes were selected, which ultimately led to a certain change in the total number of DElncRNAs.

With the continuous development of high-throughput sequencing technology, the study of lncRNAs has gradually become a research hotspot. However, as regulatory molecules, lncRNAs do not have coding ability, and their functions are typically predicted based on their associations with mRNAs. As a kind of mRNA that was found earlier and can directly guide protein biosynthesis, the function and regulatory mechanisms of mRNAs have become more mature, which can provide a reference for a more accurate understanding of the potential regulatory mechanisms of lncRNAs. Moreover, since its initial proposal, the ceRNA hypothesis has been confirmed in many studies related to drug resistance. For example, LINC00183/miR371b-5p/*Smad2* is involved in T-cell resistance to chemotherapy [41]. In this study, we used lncRNA‒mRNA association analysis to predict the functions of 276 novel DElncRNAs. The results showed that the antisense DElncRNA MSTRG.963.1 (length 1827 nt) matched three pairs of ceRNA relationship networks (MSTRG.963.1-*HCON_00005340*/*HCON_00110540* (*Rab11a*)/MSTRG.4115-miR-236-y), and the significantly downregulated target gene Rab11a was involved in two metabolic-related signalling pathways, the endocrine and other factor-regulated calcium reabsorption pathway and the vasopressin-regulated water reabsorption signalling pathway. The expression of *Rab11a*, as an important drug resistance-related gene, can inhibit cell growth, invasion and cell cycle progression [42]. In a study of resistance to gemcitabine in pancreatic cancer [43], researchers used small interfering RNA (siRNA) to verify that overexpression of *Rab11a* could increase the resistance of Capan-2 cells to gemcitabine and inhibit apoptosis induced by gemcitabine, while the absence of *Rab11a* reduced drug resistance. Further analysis revealed that MSTRG.963.1 could also bind to the target gene *HCON_00009990* (*ABCC1*) via an antisense regulatory mechanism. It is known that, ATP-binding cassette (ABC) transporters, a class of ATP-dependent transmembrane proteins, regulates the metabolism of intracellular biomass and exogenous substances by regulating the transport of molecules across the cell membrane during the generation of drug resistance. The overexpression of the ABC transporter is related to the multidrug resistance (MDR) phenotype of nematodes caused by various anthelmintics and is involved in drug excretion [44–48]. ATP-Binding Cassette Subfamily C Member 1 (*ABCC1*), a member of the ABC family, is closely related to the drug resistance of thiamethoxam (NEO), lambda-cyhalothrin (PYR), sertraline, fluoxetine and citalopram [49, 50]. In this study, the expression of MSTRG.963.1 in ABZ-resistant strains tended to be downregulated, with low transcript abundance, the expression abundance was low, and the expression of the targeted gene *ABCC1* also exhibited the same trend. Therefore, a decrease in drug-related protein efflux may be an important factor affecting albendazole resistance. Interestingly, *ABCC1* can also target 50 DElncRNAs, such as MSTRG.1643.2 and MSTRG.4812.2, through *trans*-regulatory mechanisms, which further confirms that the regulation of target genes is not achieved by a single lncRNA or a single mode of action.

Collagen, as an important part of the epidermis of nematodes, is very important for nematode body morphology, movement and reproduction. *Col* is a pathogenic gene of the stratum corneum collagen of nematodes. In the study of root-knot nematodes, knockdown of *Me-col-1* did not affect larval survival rate but significantly decreased fecundity. Moreover, overexpression of *col-120* can prolong the life span of *C. elegans* and enhance its ability to recover from heat stress [51, 52]. In this study, 5 DEGs (*col-40*, *col-155*, *col-145*, and *col-122*) and 19 targeted DElncRNAs formed 22 groups of ceRNA regulatory relationship networks, among which the *col* gene was highly expressed in the drug-resistant strains. We believe that the change in the *col* gene was regulated by multiple ceRNA axes, such as MSTRG.12969.1/novel-m0639-5p/*col-40* and MSTRG.8545.1/novel-m0189-5p/*col-145*. The oviposition rate was increased by upregulation of col gene expression to achieve drug resistance inheritance, after which the *H. contortus* responded to albendazole.

In the study reported by Siddiqui [53], steroid biosynthesis, other drug metabolizing enzymes, glycerolipid metabolism, chemical carcinogenesis, drug-metabolizing cytochrome P450, glutathione metabolism, glycerophospholipid metabolism, glycolysis/gluconeogenesis, and metabolism of xenobiotics were found to be enriched. These metabolic pathways are closely related to the effects of drugs but and each signaling pathway plays different regulatory roles. It has been reported that the miRNA PC-5p-30_205949 participates in the sensitivity to triflumezopyrim by regulating the expression of the cytochrome P450 *CYP419A1* and the ATP binding cassette transporter *ABCG23* in the small brown planthopper and *Laodelphax striatellus* [54]. At the same time, the researchers found that the *P4506FV21* (*CYP6FV21*) gene is overexpressed in a clothianidin resistant strain of *Bradysia odoriphaga*, and the sensitivity of larvae to clothianidin was significantly increased after knocking down *CYP6FV21* [55]. In our research, through gene enrichment and biological pathway analysis of DElncRNA target genes, the *HCON_00073890* (*CYP2c70*) gene was shown to be associated with the cytochrome P450 pathway, a typical drug metabolic pathway, and was positively correlated with the expression of the DElncRNAs MSTRG.11540.1, MSTRG.5400.1 and MSTRG.9730.1. Cytochrome P450 2c70 (*CYP2c70*) is an important member of the P450 enzyme superfamily. The joint effects of MSTRG.11540.1 and novel-m1192-3p are widely involved in the metabolism of xenobiotics, which determines the ceRNA relationship axis that is most likely to participate in the occurrence and development of albendazole resistance.

UDP-glycosyltransferase (UGT) is an important enzyme for detoxification of the albendazole. In research related to albendazole resistance in *H. contortus*, albendazole was shown to participate in the metabolic inactivation of insect repellents and other exogenous substrates by binding to active sugars, which can effectively protect organisms from potential exogenous substances [56, 57]. According to the constructed ceRNA network, *HCON_00127680* (*UGT2B31*) targets the DElncRNAs MSTRG.12259.1 and MSTRG.12259.1 to upregulate the expression of the downstream gene *UGT2B31*. As a member of the UGT family, the expression of the *UGT2B31* gene in ABZ-resistant strains was 1.6 times greater than that in sensitive strains, and this gene was also significantly enriched in the drug resistance pathway drug metabolism-cytochrome P450 pathway. In the study of the drug resistance of *H. contortus*, all the glycosylated metabolites of anthelmintics that have been identified are conjugates of glucose or another hexose [55], further confirming the important role of UGT in the regulation of drug resistance. Moreover, only MSTRG.12259.1, which exhibited a greater difference between the ABZ-sensitive strain and both ABZ-resistant strains, is worthy of further study.

In related studies of the resistance of osteosarcoma (OS) to cisplatin (DDP) [58], LINC00641 was identified as an intergenic noncoding RNA that enhanced drug resistance via the microRNA-320d/myeloid cell leukaemia-1 axis. Similarly, when exploring naive and vemurafenib-resistant melanoma cell lines [59], high expression of the intergenic lncRNA U73166 can be used as a biomarker of vemurafenib resistance. Therefore, intergenic lncRNAs have shown increased regulatory potential in the process of drug resistance. Analysis of the DElncRNA types between the ABZ-sensitive and ABZ-resistant samples revealed six different lncRNA types, and the number of intergenic lncRNAs accounted for 50.72% of the total DElncRNAs (95 upregulated and 45 downregulated lncRNAs, with an average length of 3,667 nt). According to the differences in expression, two intergenic lncRNAs, MSTRG.12969.2 and MSTRG.9827.1 (upregulated 12.3-fold and downregulated 11.6-fold), were screened from the ABZ-sensitive and ABZ-resistant groups. Multiple target genes were regulated by *cis*-regulatory and *trans*-regulatory pathways, and the target genes *HCON_00056550* (*gst-7*), *HCON_00096530* (*HSP20*) and *HCON_00099610* (*ABCD2*) were significantly enriched in the drug metabolism-cytochrome P450, protein processing in endoplasmic reticulum, and ABC transporter pathways. In addition, we found that MSTRG.9782.1 regulates the expression of *HCON_00192290* (*Gsk3a*) by binding to the miRNA novel-m0311-3p. *GSK3* has been shown to be involved in the resistance of AML cells to AC220, and the mechanism of drug resistance is mediated by the reactivation of downstream FGF/Ras/ERK and Wnt signal transduction [60]. In view of the high conservation of lncRNAs in nematodes [61], the intergenic lncRNAs MSTRG.12969.2 and MSTRG.9827.1 can be used as drug resistance markers in subsequent functional tests to further explore the regulatory mechanisms of ABZ resistance in *H. contortus*. Furthermore, since functional testing and/or gene silencing are still difficult in *H. contortus* [62], some predictions based on our results is useful, especially in terms of potential molecules that regulate resistance.

## Conclusions

In sum, this study provides complete data on the lncRNA expression profiles of ABZ-sensitive and ABZ-resistant strains of *H. contortus* for the first time. In the process of drug resistance development in *H. contortus*, many lncRNAs undergo significant changes in expression levels and participate in the regulation of drug resistance genes. The molecular interactions and functions of the identified drug resistance-related lncRNAs need to be further verified.

## Methods

### Sample collection

ABZ-sensitive strains: Infectious third-stage larvae were donated by Professor Wang Rui of Inner Mongolia Agricultural University. ABZ-resistant strains were collected from Wulanhot City, Inner Mongolia Autonomous Region.

The sensitivity of *H. contortus* to albendazole was verified by the faecal egg count reduction test (FECRT) and egg hatch assays (EHA), developed by the World Association for the Advancement of Veterinary Parasitology (WAAVP) [63].

FECRT: This test provides an estimate of ABZ efficacy by comparing worm egg counts from animals before and after treatment. The reduction in the FECR was calculated according to the following formula:

FECR (%) = (EPGpretreatment − EPGposttreatment)/EPGpretreatment × 100%

Resistance was considered present if the percentage reduction in egg count was less than 95% and the 95% confidence interval did not exceed 90%.

EHA: In this test, the ability of ABZ to inhibit the embryonation and hatching of freshly collected nematode eggs was evaluated by calculating the 50% lethal dose (LD_50_) of the drug. A sample was considered resistant when the LD_50_ was equal to or greater than 0.1 µg/ml.

A total of six sheep without parasites at 8 months old were transported from the pasture to the pens of the College of Veterinary Medicine, Inner Mongolia Agricultural University, and treated with closantel sodium (oral administration, at a dosage of 0.2 ml/kg), and each sheep was housed in a single pen. After five days, faecal samples were collected and examined by the McMaster technique. After twenty days, all the animals were negative for nematode eggs (the average faecal egg count was 0 eggs/gram). Six sheep were infected with ABZ-sensitive and ABZ-resistant infectious third-stage larvae (8,000 L3s/sheep, three sheep in each group). All animal procedures conducted in the present study were granted approval by the Animal Care and Use Ethics Committee of Inner Mongolia Agricultural University (Permit code: NND2023068) and Animal Ethics Guidelines from the People’s Republic of China, and all methods strictly obeyed the Guide for the Animal Research: Reporting of In Vivo Experiments (ARRIVE) guidelines 2.0. Animals had free access to food and water, while being exposed to natural lighting conditions. On day 45 after the infection, the sheep were sacrificed by intravenous administration of pentobarbital sodium to minimize suffering. Subsequently, *H. contortus* adults were isolated from the sheep abomasum, and male and female worms were identified, packed and frozen in liquid nitrogen until use for RNA extraction.

### RNA extraction, strand-specific library construction and sequencing

Samples of ABZ-sensitive and ABZ-resistant *H. contortus* were selected. A total of 60 adults were evenly distributed across two groups, with each group consisting of 30 females and 30 males. Additionally, three biological replicates were analysed from each group. Total RNA was extracted using TRIzol reagent kit (Invitrogen, Carlsbad, CA, USA) according to the manufacturer’s protocol from a total of 60 worms homogenized using a speed mill with 1 ml of TRIzol. The resulting mixture was then incubated on ice for 5 min. Afterwards, 200 µl of chloroform was added, the mixture was vortexed for 15 s, and the mixture was incubated at room temperature for 10 min, followed by centrifugation for 15 min at 12,000 g and 4 °C. The upper phase was removed, and the volume was determined. To precipitate the RNA, an equal volume of isopropanol was added, and the mixture was incubated on ice for 1 h at 4 °C, followed by centrifugation at12,000 × g for min. The supernatant was collected, washed with 1 ml of ice-cold 75% ethanol (configured in RNase-free water), air-dried and resuspended in 20 µl of RNase- free water. RNA quality was assessed on an Agilent 4200 Bioanalyzer (Agilent Technologies, Palo Alto, CA, USA) and checked using RNase-free agarose gel electrophoresis.

After total RNA was extracted, the rRNAs were removed to retain mRNAs and ncRNAs. The enriched mRNAs and ncRNAs were processed into short fragments by using fragmentation buffer and reverse transcribed into cDNA with random primers. Second-strand cDNA was synthesized by DNA polymerase I, RNase H, dNTPs (dUTP instead of dTTP) and buffer. Next, the cDNA fragments were purified with a QiaQuick PCR extraction kit (Qiagen, Venlo, The Netherlands), end repaired, poly(A) tailed, and ligated to Illumina sequencing adapters. Then, UNG (uracil-N-glycosylase) was used to digest the second-strand cDNA. The digested products were size selected by agarose gel electrophoresis, PCR amplified, and sequenced using an Illumina HiSeq™ 4000 by Gene Denovo Biotechnology Co. (Guangzhou, China).

### Quality control and read mapping

The quality evaluation and credibility analysis of the raw reads off the platform were carried out by using fastp [64]. The clean data were obtained by removing low-quality reads containing more than 10% poly(N) or more than 50% low-quality (Q-value ≤ 20) bases and adapter reads from the raw data. The short read alignment tool Bowtie2 [65] (version 2.2.8) was used for mapping reads to the ribosomal RNA (rRNA) database. The rRNA-mapped reads were subsequently removed. The remaining reads were further used in the assembly and analysis of the transcriptome. An index of the reference genome was built, and paired-end clean reads were mapped to the reference genome of *H. contortus* (GenBank: GCA_000469685.2) using HISAT2 [66].

### LncRNA prediction and expression profile analysis

Three software programs, CNCI [33], CPC [67] (http://cpc.cbi.pku.edu.cn/) and FEELNC [68] (https://github.com/tderrien/FEELnc), were used to assess the protein-coding potential of the transcripts. The intersection of the two sets of transcripts found to have no protein-coding potential was chosen as the lncRNAs. The number of lncRNA transcripts was calculated according to the position of the lncRNA relative to the protein-coding gene in the genome. The mapped reads of each sample were assembled by using StringTie v1.3.1 via a reference-based approach. For each transcription region, a fragment per kilobase of transcript per million mapped reads (FPKM) value was calculated to quantify the expression abundance and variation of the region using RSEM software [69]. The mean value of three samples in the ABZ-sensitive and ABZ-resistant treatment groups was taken as the FPKM value of the sample. The Pearson correlation coefficient between two samples was calculated based on the expression of the lncRNAs and is displayed in the form of a heatmap. The differential expression of the mRNAs and lncRNAs between the two different groups was analysed with DESeq2 software [70]. DESeq2 software was used to standardize the read counts obtained from lncRNA expression level analysis and calculate p values. Multiple hypothesis test correction was subsequently carried out to obtain the FDR value. Candidate lncRNAs were identified as those with a |log2Foldchange|≥ 1 and an FDR < 0.05, and a volcano map and clustering heatmap were generated to visualize the differences between the samples.

### lncRNA‒mRNA association analysis

To reveal the interactions between antisense lncRNAs and mRNAs, the software package RNAplex (version 0.2) [71] was used to predict the complementary associations between antisense lncRNAs and mRNAs. The program contains the Vienna RNA package, and the best base pairing was predicted based on the calculation of the minimum free energy through thermodynamic structure. LncRNAs less than 10 kb upstream or downstream of a gene may be *cis*-regulators that participate in transcriptional regulation; therefore, the DElncRNAs in this region were selected as predicted *cis*-regulated genes. In addition, the correlation between the expression of lncRNAs and protein-coding genes among samples was calculated, and the *trans*-regulatory abilities of the lncRNAs were predicted based on the Pearson correlation coefficient (absolute correlation greater than 0.95).

According to the complementary pairing of lncRNAs and mRNAs (antisense analysis), the regulation of the transcription of protein-coding genes by lncRNAs (*cis*-regulatory), and the correlation analysis of protein-coding gene and lncRNA expression (*trans*-regulatory), the relationships between lncRNAs and mRNAs was revealed by comparison with the reference genome. To better evaluate the potential roles of these DElncRNAs, functional analyses of these lncRNAs were performed with reference to the GO and KEGG databases.

### Construction of ceRNA regulatory networks based on DElncRNAs

mRNAs and lncRNAs compete with each other to bind common miRNAs through common miRNA response elements (MREs), thus regulating the expression of target gene transcripts. Based on the relationships within the ceRNA control network, a lncRNA‒miRNA–mRNA regulatory network was constructed based on the DElncRNAs (|log2Foldchange|≥ 1 and FDR < 0.05). By using a hypergeometric cumulative distribution function test, ceRNA relationship pairs with *P* < 0.05 were selected and Cytoscape [72] was used to construct a ceRNA regulatory network diagram.

### CeRNA connectivity analysis in drug-resistant pathways

Pathway-RNA connectivity network visualization analysis was performed using Cytoscape software for all key RNAs enriched in resistance-related pathways. The RNA molecules and hub genes strongly related to albendazole resistance in *H. contortus* were found.

#### qRT‒PCR

Six lncRNAs and six mRNAs identified as differentially expressed were selected, and primers were designed with Oligo7 based on the lncRNA sequences (Additional file 1: Table S1) to ensure specificity and avoid overlap with the target genes. The RNA used for qRT‒PCR was the same as that used for sequencing. The lncRNA and mRNA sequences were amplified using the PrimeScript™ RT Reagent Kit with gDNA Eraser (Perfect Real Time), and qRT–PCR was subsequently performed using the TB Green® Premix EX Taq™ II (Tli RNaseH Plus) Kit according to the manufacturer’s protocol. All qRT‒PCR was carried out in an Applied Biosystem QuantStudio 7 Flex Real-time PCR machine (Thermo Fisher Scientific, USA) in reaction mixtures of 10 µL of TB Green premix EX Taq II, 1.0 µL of diluted cDNA, 7.4 µL of RNase-free water, and 0.8 µL of each 10 µM primer. The DElncRNAs and DEmRNAs were measured by qRT‒PCR with glyceraldehyde-3-phosphate dehydrogenase (*GAPDH*) serving as the internal reference gene, and three replicates were performed for each group. The expression level of the target gene relative to the internal reference gene in each sample was calculated according to the 2^−∆∆Ct^ method and compared with the results of transcriptome sequencing for verification. Statistical significance was determined by using Student’s t test in GraphPad Prism Version 8.0.2. Quantitative data are presented as the means ± SDs, and the significance level for the statistical analysis was set as *P* < 0.05.

### Supplementary Information

**Supplementary material 1. **

## Data Availability

The datasets of this article are included within the manuscript and its supplementary materials. The RNA-seq raw data were submitted to the National Centre for Biotechnology Information (NCBI) under the bio-project number PRJNA971145 (https://www.ncbi.nlm.nih.gov/bioproject/PRJNA971145).

## Abbreviations

ABZ: Albendazole
*H.contortus*: *Haemonchus contortus*
lncRNAs: Long noncoding RNAs
DElncRNAs: Differentially expressed lncRNAs
GO: Gene Ontology
KEGG: Kyoto Encyclopedia of Genes and Genomes
ceRNA: Competitive endogenous RNA
SNP: Single nucleotide polymorphism
qRT-PCR: Quantitative real-time polymerase chain reaction
P-gps: P-glycoprotein encoding genes
ncRNA: Non-coding RNA
mRNA: Messenger RNA
rRNA: Ribosome RNA
FPKM: Fragment per kilobase of transcript per million mapped reads
MRE: MiRNA response elements
GAPDH: Glyceraldehyde-3-phosphate dehydrogenase
FDR: False discovery rate
FECRT: Faecal egg count reduction test
EHA: Egg hatch assays
WAAVP: World Association for the Advancement of Veterinary Parasitology
DEGs: Different expressed genes
MDR: Multidrug resistance
ABC: ATP-binding cassette transporter
UGT: UDP-Glycosyltransferase
