# Supplementary material for: Transcriptome reveals the roles and potential mechanisms of lncRNAs in the regulation of albendazole resistance in Haemonchus contortus
Source: BMC Genomics. 2024 Feb 17;25:188. doi: 10.1186/s12864-024-10096-6 (PMC10873934; doi:10.1186/s12864-024-10096-6)
Supplement: Supplementary file 1 — Supplementary material 1. [file 12864_2024_10096_MOESM1_ESM.pdf]

Supplementary Information

Transcriptome reveals the role and potential mechanism of lncRNAs in the regulation of albendazole resistance in *Haemonchus contortus*

Xindi Chen[1], Tengyu Wang [1], Wenrui Guo [1], Xu Yan [1], Huilin Kou1, Yu Yu [1], Chunxia Liu[2], Wa Gao[3], Wenlong Wang[1*], Rui Wang[1*]

[1] Key Laboratory of Animal Disease Clinical Diagnosis and Treatment Technology, College of Veterinary Medicine, Inner Mongolia Agricultural University, Hohhot, 010018, China

[2] Key Laboratory of Animal Disease Clinical Diagnosis and Treatment Technology, College of Life Science, Inner Mongolia Agricultural University, Hohhot, 010018, China

[3] Inner Mongolia Key Laboratory of Tick-Borne Zoonotic Infectious Disease, Department of Medicine, Hetao College, Bavan Nur 015000, China

[*]Correspondence: wwl.imau@163.com; wr2006@163.com

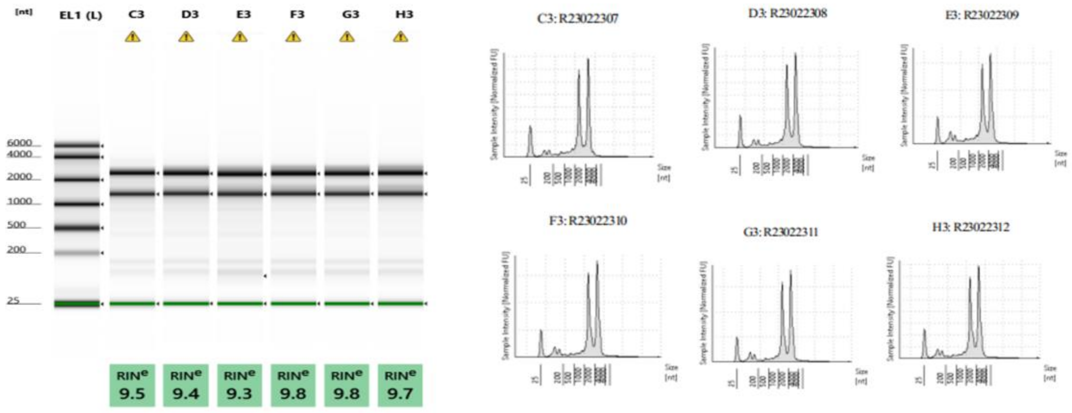

Figure S1. Quality Control of RNA-seq. C3, ABZ-S1. D3, ABZ-S2. E3, ABZ-S3. F3, ABZ-R1. G3, ABZ-R2. H3, ABZ-R3. Detection of RNA gel electrophoresis. ALL RNAs Integrity Number > 9).

Table S1. Primer sequences used for qRT-PCR analysis

**lncRNA ID**	**Primer(5′ to 3′)**
MSTRG.3773.1	ACATGAGCCGTTCGAGAGTG
	ACGGCGAGGCAGTATTATAGC
MSTRG.3773.2	GAGTGGCGGCTGTGATTGT
	TAATAAACGGCGAGGC
MSTRG.7803.1	AACCTGCAAATTCCCACAGG
	TTAACGACCCAAACCGCCTA
MSTRG.6845.2	CTACTCGCCTTTGCCATGCTC
	TCTCTTCGGAATTCTTTGTGCTGA
MSTRG.7878.1	CCTGTGGAATTGCCAGAAGAT
	CACTCTTTTGACCGGGAATCG
MSTRG.1168.2	CCGCATGTATTTTATCTGACC
	ATCGCTCTATAAAACATGGAC
HCON_00009990	ATGGCTTCATTGATTCCGTTC
	AAGCGTAAAGTTTAACCACCT
HCON_00189670	CTTCATTAATTTGTGCTCCCT
	TTCAAACCACCGAAGATCGT
HCON_00141050	CTTCAATGGGCTCACTACCAA
	ATGCTTCGTAATTCCTCGTTG
HCON_00141020	ATTTCTCGCTACCTTTACTGCT
	GGCCTTTTCTTTCCATCAGACT
HCON_00124060	GTAAAATCACGTTACGCTCA
	TTGTCGGAACATAAACAAGC
HCON_00085780	AAAGGTCCAAAAGTACGCTCT
	GACCTCTACTCTCATTCTGTGCT
GAPDH	TGCTGTCGAGAAGGACACTG
	ATCCTGTGAGTGCTCTTGCC

Table S2. Quality metrics of the clean reads

Samples	RawData (bp)	BF_Q20 (%)	BF_Q30 (%)	BF_N (%)	BF_GC (%)	CleanData (bp)	AF_Q20 (%)	AF_Q30 (%)	AF_N (%)	AF_GC (%)
ABZ-S1	11403175500	10889582406 (95.50%)	10140867684 (88.93%)	664704 (0.01%)	5264061703 (46.16%)	11288579337	10800161128 (95.67%)	10063135011 (89.14%)	243173 (0.00%)	5207148486 (46.13%)
ABZ-S2	11219175600	10649735611 (94.92%)	9881954181 (88.08%)	623638 (0.01%)	5183943084 (46.21%)	11053462313	10517469181 (95.15%)	9766973236 (88.36%)	241729 (0.00%)	5102231756 (46.16%)
ABZ-S3	11560346400	10981052320 (94.99%)	10191907082 (88.16%)	658363 (0.01%)	5383382188 (46.57%)	11410722181	10862967985 (95.20%)	10089486231 (88.42%)	238683 (0.00%)	5308291868 (46.52%)
ABZ-R1	11497460700	10927537260 (95.04%)	10147445650 (88.26%)	653267 (0.01%)	5331627961 (46.37%)	11348179587	10809241716 (95.25%)	10044819485 (88.51%)	252795 (0.00%)	5256212625 (46.32%)
ABZ-R2	11372158800	10814415204 (95.10%)	10041828336 (88.30%)	644498 (0.01%)	5244166330 (46.11%)	11234069542	10704926366 (95.29%)	9946772284 (88.54%)	232824 (0.00%)	5174891637 (46.06%)
ABZ-R3	11151021000	10604829163 (95.10%)	9847076885 (88.31%)	636212 (0.01%)	5163666570 (46.31%)	11006366269	10488699705 (95.30%)	9745857860 (88.55%)	229478 (0.00%)	5090537094 (46.25%)

Samples: Sample group names

ABZ-SX: ABZ-sensitive strain X

ABZ-RX: ABZ-resistant strain X

RawData: Original reads number

CleanData: The reads number after filtering

AF: After filter

BF: Before filter

Q20 (%): The number of bases whose sequencing base quality value reaches the level above Q20 and the percentage of RawData (or CleanData)

Q30 (%): The number of bases whose sequencing base quality value reaches the level above Q20 and the percentage of RawData (or CleanData)

N (%): The number of N bases contained in single-ended reads and the percentage of RawData (or CleanData)

GC (%): Sequence base GC ratio before (after) filtering

Table S3. Information on DElncNRNAs between ABZ-senstive and ABZ-resistant strains of *Haemonchus contortus*.

	**LncRNA ID**	**ABZ-sensitive FPKM mean**	**ABZ-resistant FPKM mean**	**log2(fc)**	**PValue**	**FDR**
Up-regulated (175)	MSTRG.12969.2	0.001	5.060333333	12.30501671	4.46E-07	4.69E-06
	MSTRG.9782.1	0.001	4.049666667	11.98358745	1.17E-07	1.36E-06
	MSTRG.11528.1	0.001	3.015	11.55794229	2.14E-06	1.99E-05
	MSTRG.1298.2	0.001	1.924333333	10.91014301	7.45E-05	0.000520579
	MSTRG.444.1	0.001	1.588	10.6329952	0.000299183	0.00181765
	MSTRG.2289.1	0.001	1.532	10.58120058	8.25E-05	0.00056969
	MSTRG.2827.1	0.001	1.426666667	10.47843258	0.000947457	0.004980326
	MSTRG.10000.1	0.001	1.405	10.45635442	0.008476554	0.031630104
	MSTRG.8774.1	0.001	1.054666667	10.04257138	0.003466834	0.015186241
	MSTRG.1718.1	0.001	1.033	10.01262454	0.000553935	0.003086208
	MSTRG.1006.1	0.001	1.019333333	9.993410191	0.005137608	0.020773808
	MSTRG.3696.1	0.001	0.968333333	9.919359948	0.000434376	0.00245402
	MSTRG.11916.1	0.001	0.897333333	9.809500194	0.001844387	0.009101485
	MSTRG.3038.1	0.001	0.804333333	9.651649699	0.012380163	0.043510515
	MSTRG.721.1	0.001	0.622	9.28077077	0.009303516	0.033981724
	MSTRG.11918.1	6.501333333	663.1786667	6.672518163	2.61E-104	7.89E-102
	MSTRG.11914.2	1.399	126.8993333	6.503144717	1.93E-65	3.33E-63
	MSTRG.11914.7	14.02066667	870.6436667	5.956455525	9.68E-123	1.17E-119
	MSTRG.8581.1	1.757666667	107.1796667	5.930225928	1.41E-82	3.41E-80
	MSTRG.3343.1	0.488666667	29.74266667	5.927539494	0.000905087	0.004799343
	MSTRG.11914.8	18.194	1008.324667	5.792353673	4.68E-74	9.44E-72
	MSTRG.11913.1	0.789666667	43.72866667	5.791191757	5.50E-34	3.70E-32
	MSTRG.9781.1	0.243333333	11.00666667	5.499299846	7.96E-13	1.72E-11
	MSTRG.5764.1	0.044333333	1.981333333	5.481935965	0.000141148	0.000907702

MSTRG.2377.1	0.869	37.28266667	5.423005066	1.54E-20	5.16E-19
MSTRG.11914.4	7.252333333	310.4076667	5.419575144	1.82E-116	1.10E-113
MSTRG.2181.1	0.258666667	10.259	5.309652149	2.21E-11	3.87E-10
MSTRG.1443.1	1.568	54.88766667	5.129484546	1.41E-64	2.14E-62
MSTRG.1289.1	0.029333333	0.982333333	5.065599726	0.000944324	0.004980326
MSTRG.8563.1	0.128	4.127	5.010877723	7.90E-07	7.89E-06
MSTRG.9436.2	0.089333333	2.700333333	4.917795102	8.14E-08	9.56E-07
MSTRG.4720.1	0.03	0.891	4.892391026	0.0016856	0.008386382
MSTRG.4410.1	0.048666667	1.343	4.786381531	0.004315381	0.017990459
MSTRG.8772.1	0.057666667	1.529	4.728706964	0.003708367	0.015955218
MSTRG.11758.1	0.068333333	1.740333333	4.670630344	0.004187556	0.017640263
MSTRG.8541.1	0.053333333	1.335666667	4.6463787	0.010230059	0.036906028
MSTRG.2379.1	0.409666667	10.081	4.621044436	1.88E-11	3.35E-10
MSTRG.12969.1	0.349	8.481666667	4.603048844	8.90E-10	1.33E-08
MSTRG.3003.1	0.337	8.121333333	4.590896107	1.34E-20	4.62E-19
MSTRG.3313.2	3.368	80.499	4.579006818	5.76E-46	5.81E-44
MSTRG.11328.1	0.164333333	3.762666667	4.517058436	2.41E-05	0.000188216
MSTRG.7523.1	0.725	16.06166667	4.469496799	9.22E-09	1.22E-07
MSTRG.3051.1	1.081	23.77766667	4.45916872	2.10E-23	8.47E-22
MSTRG.7318.1	3.011	65.06333333	4.433530123	2.39E-33	1.52E-31
MSTRG.1298.1	0.140666667	2.752	4.290128067	0.0002451	0.001511866
MSTRG.3255.1	0.549666667	10.722	4.285873237	6.33E-13	1.39E-11
MSTRG.8545.1	0.074666667	1.418666667	4.247927513	0.013458844	0.046490693
MSTRG.3312.2	2.407	44.67166667	4.214052035	1.17E-23	4.87E-22
MSTRG.1010.2	0.208666667	3.768	4.174526903	1.24E-09	1.80E-08
MSTRG.1076.1	2.174666667	39.04	4.166086866	1.06E-46	1.28E-44
MSTRG.8580.1	1.218	21.69366667	4.15468788	6.95E-19	2.10E-17

MSTRG.3345.1	1.299	21.96766667	4.079908304	2.40E-22	9.37E-21
MSTRG.7820.1	0.121333333	2.041333333	4.072463927	0.007813688	0.029613633
MSTRG.5313.1	0.933333333	15.559	4.059213108	2.05E-17	5.75E-16
MSTRG.2710.1	0.892666667	14.072	3.978562023	2.92E-22	1.10E-20
MSTRG.11914.6	0.213	3.155	3.88871467	0.000329896	0.001969306
MSTRG.3256.1	0.515333333	7.610666667	3.884445016	1.35E-07	1.55E-06
MSTRG.1407.1	58.93833333	865.177	3.875717144	1.91E-111	7.69E-109
MSTRG.3272.1	0.765	11.06533333	3.854443354	1.58E-07	1.81E-06
MSTRG.3274.1	0.290333333	4.124333333	3.828378814	2.48E-06	2.27E-05
MSTRG.2342.1	9.505333333	133.772	3.814895145	6.85E-18	1.97E-16
MSTRG.10779.1	2.773666667	38.99766667	3.813521483	2.65E-33	1.60E-31
MSTRG.3055.1	0.291333333	4.012666667	3.783818632	9.74E-06	8.01E-05
MSTRG.5968.1	0.728333333	9.951333333	3.77221906	9.46E-12	1.82E-10
MSTRG.10638.1	0.776666667	10.50366667	3.757453679	3.16E-09	4.39E-08
MSTRG.10538.3	0.931666667	12.23366667	3.714899185	1.78E-11	3.21E-10
MSTRG.10779.2	1.390333333	18.19866667	3.710330038	1.05E-11	1.95E-10
MSTRG.3257.1	0.085	1.105	3.700439718	0.008594001	0.031894674
MSTRG.7325.1	2.183	27.085	3.633110055	7.37E-29	4.05E-27
MSTRG.12598.2	1.874	23.03433333	3.619592986	7.36E-17	1.93E-15
MSTRG.2467.1	0.671666667	8.215	3.612443237	5.89E-10	8.90E-09
MSTRG.8984.1	0.463333333	5.451666667	3.556574971	4.59E-08	5.66E-07
MSTRG.12986.1	1.831	21.47933333	3.55224552	2.39E-13	5.55E-12
MSTRG.11903.1	9.335333333	109.489	3.551940587	3.22E-38	2.59E-36
MSTRG.7693.1	0.174666667	1.927333333	3.463929892	0.002712371	0.012281859
MSTRG.8580.2	0.505666667	5.503	3.443959744	7.70E-07	7.83E-06
MSTRG.6336.1	0.224333333	2.417	3.429501564	0.000310478	0.001876841
MSTRG.3308.1	1.114666667	11.99433333	3.42766872	8.57E-11	1.38E-09

MSTRG.7322.1	2.933333333	30.13533333	3.360843095	4.75E-20	1.51E-18
MSTRG.12259.1	0.166666667	1.620666667	3.281549893	0.002554829	0.011744441
MSTRG.7834.1	0.268666667	2.570333333	3.258066224	0.00035252	0.002089201
MSTRG.5755.2	2.347	22.381	3.253384751	4.00E-17	1.08E-15
MSTRG.4742.1	0.242333333	2.263666667	3.223596763	0.003955885	0.016840371
MSTRG.5356.1	0.171333333	1.566333333	3.192513503	0.003205664	0.014248704
MSTRG.2454.1	0.837666667	7.456666667	3.15408268	1.38E-06	1.30E-05
MSTRG.9370.2	3.628333333	32.208	3.150040171	5.50E-14	1.36E-12
MSTRG.8077.1	0.203666667	1.798333333	3.142378675	0.006240695	0.024576546
MSTRG.7319.1	10.09733333	87.401	3.113675453	4.19E-12	8.56E-11
MSTRG.10660.1	0.366666667	3.140666667	3.098529808	0.006852344	0.026467999
MSTRG.9784.1	2.321	19.74966667	3.089009876	1.22E-11	2.24E-10
MSTRG.8577.1	17.738	139.8876667	2.979353515	1.30E-42	1.21E-40
MSTRG.1409.1	54.59966667	430.3133333	2.978423495	2.66E-42	2.30E-40
MSTRG.7324.1	0.163	1.280333333	2.973575594	0.0009522	0.004983592
MSTRG.3259.1	4.518	33.755	2.901345043	4.25E-12	8.56E-11
MSTRG.7238.4	18.22566667	132.358	2.860401905	1.92E-62	2.58E-60
MSTRG.11545.1	0.312666667	2.211666667	2.822436638	0.011443112	0.040334468
MSTRG.1010.1	0.259	1.789	2.788129384	1.10E-05	8.95E-05
MSTRG.9919.1	0.208	1.422	2.773266031	9.38E-05	0.000640668
MSTRG.9371.1	7.737	52.007	2.74885964	0.000327112	0.001967556
MSTRG.6008.3	0.902666667	6.066666667	2.748638806	2.60E-05	0.000200501
MSTRG.13012.1	2.386333333	15.71266667	2.719060564	2.55E-10	4.00E-09
MSTRG.3658.1	0.583666667	3.718666667	2.671568851	0.00040879	0.00233126
MSTRG.8021.1	0.677666667	4.266333333	2.654348975	0.001881523	0.009209562
MSTRG.2459.1	3.77	23.46333333	2.637771557	3.55E-05	0.000263484
MSTRG.15648.1	0.245666667	1.517333333	2.626764033	0.006554724	0.025619467

MSTRG.1168.2	8.508333333	52.062	2.613282275	8.84E-25	3.82E-23
MSTRG.11841.1	12.831	77.80133333	2.600161267	1.27E-36	9.00E-35
MSTRG.11920.1	0.676666667	4.052333333	2.582235624	1.17E-06	1.13E-05
MSTRG.13012.2	2.082333333	12.46633333	2.58176426	2.82E-07	3.15E-06
MSTRG.746.2	0.429333333	2.565666667	2.579163654	0.00114932	0.005862987
MSTRG.3348.1	14.83666667	88.05533333	2.569243388	3.29E-16	8.47E-15
MSTRG.9799.1	0.638333333	3.710666667	2.539296517	0.005455456	0.021912447
MSTRG.925.1	18.714	108.279	2.532563605	3.86E-22	1.41E-20
MSTRG.9436.5	0.639333333	3.682	2.525849407	0.004278437	0.017898373
MSTRG.9370.1	7.302333333	41.92733333	2.521461644	2.84E-06	2.58E-05
MSTRG.7323.1	34.566	196.8476667	2.509654043	7.01E-14	1.70E-12
MSTRG.11920.3	487.8256667	2694.379	2.465515227	5.63E-27	2.84E-25
MSTRG.2315.1	4.732666667	26.046	2.46033661	3.73E-11	6.36E-10
MSTRG.8495.1	9.119	49.86433333	2.451060731	2.20E-17	6.05E-16
MSTRG.7238.3	3.215666667	17.451	2.440119939	4.04E-10	6.26E-09
MSTRG.7323.2	47.70166667	256.6076667	2.427452695	4.49E-14	1.13E-12
MSTRG.5276.1	0.372	1.947	2.387878358	0.008112152	0.03036406
MSTRG.10134.2	51.94933333	269.805	2.376739944	8.90E-26	4.14E-24
MSTRG.579.1	1.165666667	5.901333333	2.339885655	2.74E-05	0.000209293
MSTRG.7514.1	127.1483333	634.7096667	2.319584264	1.75E-26	8.46E-25
MSTRG.3158.1	1.214	5.810333333	2.25885251	9.48E-05	0.000644179
MSTRG.7516.1	7.540333333	35.767	2.245928909	3.96E-11	6.65E-10
MSTRG.2290.1	1.024666667	4.592333333	2.164072699	0.000121776	0.000791546
MSTRG.11438.1	2.009	8.914333333	2.149649345	7.98E-12	1.58E-10
MSTRG.1604.1	0.876666667	3.876333333	2.144592337	0.002138744	0.010230909
MSTRG.145.1	0.660666667	2.882666667	2.125409561	0.000680278	0.003688143
MSTRG.7821.1	1.969333333	8.529	2.114669274	0.000454354	0.002543121

MSTRG.13010.1	3.002	12.962	2.110292457	1.52E-05	0.000121898
MSTRG.3204.2	0.497666667	2.142666667	2.106155764	0.014122737	0.048644982
MSTRG.3346.1	3.769333333	16.12266667	2.096709096	1.01E-05	8.28E-05
MSTRG.3258.1	2.716333333	11.37766667	2.066472285	5.43E-05	0.000390479
MSTRG.9373.1	33.13366667	138.3503333	2.06195635	2.92E-25	1.31E-23
MSTRG.3346.2	6.112666667	25.317	2.050232657	1.58E-06	1.49E-05
MSTRG.13449.1	10.869	44.274	2.02624051	3.49E-07	3.84E-06
MSTRG.3346.3	14.59433333	58.819	2.010873946	5.19E-09	7.05E-08
MSTRG.9730.1	5.566	22.16033333	1.993266769	2.66E-09	3.78E-08
MSTRG.7396.1	1.928666667	7.613333333	1.980924422	7.22E-06	6.23E-05
MSTRG.12839.1	1.797333333	6.977666667	1.956886684	0.000390873	0.002261077
MSTRG.12741.1	9.925666667	37.90366667	1.933101506	6.31E-07	6.46E-06
MSTRG.3422.3	4.013333333	15.125	1.914062251	7.78E-06	6.63E-05
MSTRG.11902.1	3.067333333	11.452	1.900542715	6.97E-08	8.34E-07
MSTRG.5400.1	0.469666667	1.749333333	1.897096108	0.000590129	0.003257833
MSTRG.14739.1	0.808	2.993333333	1.889325746	4.17E-05	0.000307275
MSTRG.6785.1	5.247	18.787	1.840170013	1.83E-13	4.33E-12
MSTRG.3077.2	8.198666667	29.15333333	1.830199636	0.000561558	0.003114331
MSTRG.8331.1	1.993666667	7.012666667	1.814538937	4.79E-10	7.33E-09
MSTRG.1531.2	1.025333333	3.571666667	1.800504442	0.0019838	0.009632183
MSTRG.14070.1	0.622333333	2.164333333	1.798163282	0.000670932	0.003653862
MSTRG.8499.3	23.88633333	82.483	1.787911396	8.30E-09	1.11E-07
MSTRG.8885.2	52.413	170.9093333	1.705234592	5.57E-13	1.25E-11
MSTRG.9372.1	138.0926667	409.9326667	1.569750252	3.01E-09	4.23E-08
MSTRG.10174.3	0.684333333	2.016	1.558724512	0.013052894	0.045741881
MSTRG.1577.1	1.903666667	5.304666667	1.478481214	0.000137321	0.00088781
MSTRG.924.1	7.943666667	21.92466667	1.464677918	3.32E-07	3.69E-06

	MSTRG.12840.1	3.514333333	9.684	1.462352042	0.002565817	0.011750275
	MSTRG.404.1	2.517	6.920333333	1.459136314	0.010779928	0.038332157
	MSTRG.501.1	10.63666667	27.64466667	1.377955068	2.45E-12	5.11E-11
	MSTRG.10423.1	2.273333333	5.906333333	1.377453541	0.001431383	0.007210592
	MSTRG.13859.1	19.58433333	48.88566667	1.319711509	7.35E-08	8.71E-07
	MSTRG.11699.1	30.48266667	71.61033333	1.232178665	7.62E-06	6.53E-05
	MSTRG.11540.1	0.985333333	2.265666667	1.201251853	0.004557927	0.018743314
	MSTRG.9376.1	62.298	142.0096667	1.188731385	0.000330661	0.001969306
	MSTRG.8762.3	4.043666667	9.043333333	1.161190566	0.000801036	0.00428519
	MSTRG.4693.1	3.680666667	8.227666667	1.160516246	0.004096555	0.017317257
	MSTRG.8660.2	8.648	18.51333333	1.098126248	4.36E-05	0.000319207
	MSTRG.13862.1	43.578	93.06833333	1.094690386	2.49E-08	3.20E-07
	MSTRG.10122.1	6.247666667	13.31466667	1.091626923	0.002300914	0.010740559
	MSTRG.2375.2	4.332666667	8.763666667	1.016279364	7.32E-05	0.000514545
	MSTRG.5476.1	7.359	14.831	1.011034237	0.000973552	0.005073383
	MSTRG.8578.1	59.252	118.6926667	1.002295045	4.43E-06	3.92E-05
Down-regulated (101)	MSTRG.9827.1	3.187333333	0.001	-11.63813419	2.99E-06	2.69E-05
	MSTRG.963.1	2.276	0.001	-11.15228484	9.39E-06	7.78E-05
	MSTRG.7719.1	2.168333333	0.001	-11.08237084	1.72E-07	1.94E-06
	MSTRG.871.1	1.818666667	0.001	-10.82866543	1.14E-06	1.11E-05
	MSTRG.8672.2	1.589666667	0.001	-10.63450857	1.31E-08	1.72E-07
	MSTRG.13992.1	1.217666667	0.001	-10.24990354	7.10E-05	0.000502023
	MSTRG.12193.2	1.195	0.001	-10.2227949	0.006916616	0.026631174
	MSTRG.4835.1	0.913666667	0.001	-9.835524112	0.009531527	0.034605454
	MSTRG.1620.1	0.851333333	0.001	-9.733580309	0.001390067	0.007031761
	MSTRG.13713.1	1.146666667	0.032	-5.163230349	0.005476955	0.021925956
	MSTRG.9891.1	0.783333333	0.028666667	-4.772180287	0.002176648	0.010279562

MSTRG.3269.1	1.709666667	0.081666667	-4.387823917	6.13E-05	0.000438804
MSTRG.1643.2	1.112	0.058333333	-4.252692462	0.000781811	0.004200928
MSTRG.15545.1	17.97033333	0.945333333	-4.248650233	1.18E-36	8.92E-35
MSTRG.10343.1	3.416	0.234666667	-3.863623142	6.60E-05	0.000469358
MSTRG.7300.1	2.288666667	0.186333333	-3.618549669	3.88E-08	4.86E-07
MSTRG.7620.2	0.511666667	0.042	-3.606743017	0.010309312	0.03698504
MSTRG.12262.1	2.238333333	0.198333333	-3.496425826	0.00018822	0.001172982
MSTRG.4144.1	1.059666667	0.102666667	-3.36757076	0.00943213	0.034347725
MSTRG.3269.2	1.083	0.107666667	-3.330389674	0.002541296	0.011728846
MSTRG.3477.8	5.360666667	0.544666667	-3.298966946	0.000365583	0.002156049
MSTRG.11077.2	2.726	0.305333333	-3.158328559	0.000415737	0.002359749
MSTRG.3477.7	1.369666667	0.159666667	-3.100689769	0.004690813	0.019224384
MSTRG.14618.1	1.327	0.156	-3.088550437	0.000253016	0.001552773
MSTRG.1158.1	2.243666667	0.299666667	-2.904427836	0.000367615	0.00215751
MSTRG.12481.1	4.873	0.659333333	-2.885730297	1.32E-06	1.27E-05
MSTRG.2774.1	2.020333333	0.274333333	-2.880591507	0.005847951	0.023257149
MSTRG.2387.1	1.586	0.232333333	-2.771124711	8.58E-05	0.000589516
MSTRG.1489.1	3.512	0.56	-2.648794113	0.00155252	0.007756187
MSTRG.8069.1	0.885666667	0.146666667	-2.594222799	0.00374807	0.016068852
MSTRG.12387.2	2.007	0.357333333	-2.489698212	0.000108711	0.000734253
MSTRG.7020.1	3.103	0.557666667	-2.476188752	0.004806013	0.019563871
MSTRG.10520.1	2.607	0.481	-2.438281784	0.00433021	0.017990459
MSTRG.5385.2	2.112333333	0.414333333	-2.349973719	0.000285793	0.001745071
MSTRG.15532.5	4.829	0.994666667	-2.279439429	0.000742267	0.004006253
MSTRG.8336.1	2.285	0.474666667	-2.26720752	2.43E-05	0.000188584
MSTRG.14276.2	5.673	1.245666667	-2.187193801	0.001093495	0.005625685
MSTRG.13030.1	1.775333333	0.399333333	-2.15242452	0.00297906	0.013389157

MSTRG.10307.1	2.648333333	0.610666667	-2.116627716	0.006587902	0.025619467
MSTRG.10123.1	9.101	2.113666667	-2.106277198	0.00011396	0.000761205
MSTRG.2764.2	3.728666667	0.870666667	-2.098467433	3.94E-07	4.21E-06
MSTRG.11848.1	5.000666667	1.213	-2.043540891	4.53E-05	0.000329835
MSTRG.7021.1	2.295	0.562333333	-2.028996681	0.00296825	0.013389157
MSTRG.8053.1	1.990333333	0.522666667	-1.929047009	0.00015318	0.000974712
MSTRG.7159.2	2.513333333	0.662333333	-1.923972651	0.009196015	0.03379326
MSTRG.10904.2	18.96333333	5.032	-1.914008753	1.58E-08	2.06E-07
MSTRG.4812.2	6.303	1.753333333	-1.845938364	8.31E-11	1.36E-09
MSTRG.8470.1	46.32	13.07133333	-1.825228943	1.21E-19	3.74E-18
MSTRG.2499.1	2.788	0.791666667	-1.816265548	0.005916391	0.023452186
MSTRG.7437.1	7.024333333	2.038	-1.785207257	0.002285159	0.010708363
MSTRG.12275.1	7.763666667	2.274333333	-1.771294462	1.94E-05	0.000151924
MSTRG.3596.1	1.520666667	0.452666667	-1.748182967	0.00472237	0.019288327
MSTRG.12793.1	1.934	0.586	-1.722615225	0.010890306	0.038611086
MSTRG.11368.1	3.449333333	1.051333333	-1.714097394	2.91E-05	0.00022004
MSTRG.13092.1	5.112	1.602333333	-1.673713534	0.008700227	0.032068825
MSTRG.12842.4	2.24	0.755	-1.568950183	0.00611677	0.024167238
MSTRG.642.1	0.611333333	0.207666667	-1.557689571	0.004043218	0.017151754
MSTRG.3476.5	4.324333333	1.475333333	-1.551436784	3.15E-05	0.00023511
MSTRG.10905.2	57.45466667	19.65733333	-1.547356456	1.91E-05	0.000150772
MSTRG.11067.1	1.861	0.639666667	-1.540685845	0.004213719	0.017688842
MSTRG.9633.1	8.966666667	3.085666667	-1.538989546	4.44E-06	3.92E-05
MSTRG.11391.1	2.304666667	0.805333333	-1.516900149	0.0072466	0.027725125
MSTRG.13807.2	3.139	1.107333333	-1.503215456	0.001693879	0.00839303
MSTRG.10719.1	2.098666667	0.740666667	-1.502576724	0.002541735	0.011728846
MSTRG.3492.1	12.63866667	4.494	-1.491772243	1.52E-10	2.42E-09

MSTRG.5895.1	55.852	19.86533333	-1.491355941	1.38E-28	7.26E-27
MSTRG.3477.5	7.517	2.685	-1.485234916	4.96E-11	8.22E-10
MSTRG.8654.1	2.715333333	0.982666667	-1.46635529	0.01320666	0.046013981
MSTRG.10769.3	7.056	2.554	-1.466092036	0.002160793	0.0102447
MSTRG.2293.2	4.081333333	1.501	-1.443116567	0.003664664	0.015823494
MSTRG.9271.1	2.272	0.850333333	-1.417862437	0.007210788	0.027675692
MSTRG.2505.1	1.094333333	0.411666667	-1.410503708	0.010371791	0.037099097
MSTRG.642.2	1.010666667	0.381	-1.40744435	0.000194616	0.001206621
MSTRG.11686.1	25.598	9.678333333	-1.403200562	4.70E-31	2.71E-29
MSTRG.11236.1	497.0073333	189.3273333	-1.39238443	1.41E-46	1.55E-44
MSTRG.3477.4	1.459333333	0.563	-1.374102626	0.001857271	0.009127808
MSTRG.4794.1	11.637	4.561666667	-1.351086248	1.14E-06	1.11E-05
MSTRG.1643.3	2.459666667	0.979333333	-1.32859092	0.000986508	0.005118834
MSTRG.10710.1	15.31766667	6.494666667	-1.237869161	5.94E-08	7.19E-07
MSTRG.1035.1	74.02166667	31.50333333	-1.232443133	6.09E-21	2.16E-19
MSTRG.12193.1	20.18	8.602333333	-1.230126234	0.000121265	0.000791546
MSTRG.15436.1	3.137	1.358333333	-1.207547971	0.003400084	0.014948006
MSTRG.1814.1	4.199333333	1.845	-1.186539494	0.000641298	0.003524226
MSTRG.12501.4	15.156	6.738	-1.169496711	0.000113427	0.000761205
MSTRG.15251.1	2.456666667	1.098333333	-1.161386154	0.000187088	0.001171968
MSTRG.12455.1	1.983	0.89	-1.155807436	0.003337807	0.01472777
MSTRG.11030.3	14.33166667	6.484	-1.144250398	1.36E-06	1.29E-05
MSTRG.12264.9	8.418666667	3.828666667	-1.136749699	8.90E-12	1.74E-10
MSTRG.12962.1	4.433333333	2.018	-1.135465665	0.002647623	0.012033745
MSTRG.5330.1	64.14	29.53666667	-1.118717333	3.24E-18	9.56E-17
MSTRG.5669.1	2.855666667	1.320333333	-1.112925387	0.001378343	0.00700175
MSTRG.11688.1	48.981	22.70033333	-1.109508746	4.96E-13	1.13E-11

	MSTRG.12264.1	6.08	2.830333333	-1.103099352	7.66E-05	0.000532292
	MSTRG.12308.1	9.537333333	4.517333333	-1.078114567	0.004402216	0.018219216
	MSTRG.13304.1	7.975	3.780666667	-1.076843863	0.00633868	0.024881375
	MSTRG.10122.2	6.519	3.135666667	-1.055878472	0.013423141	0.046490693
	MSTRG.959.1	30.42333333	14.76133333	-1.04335519	6.73E-06	5.85E-05
	MSTRG.12193.5	186.6036667	90.569	-1.042888101	6.21E-07	6.45E-06
	MSTRG.13723.1	21.82166667	10.699	-1.028285335	6.25E-07	6.45E-06
	MSTRG.10792.1	6.735666667	3.307	-1.026297699	0.010955987	0.038730376
	MSTRG.6208.1	2.643666667	1.318333333	-1.003825089	0.002152403	0.0102447
ABZ-sensitive expression level (top 10)	MSTRG.11236.1	497.0073333	189.3273333	-1.39238443	1.41E-46	1.55E-44
	MSTRG.11920.3	487.8256667	2694.379	2.465515227	5.63E-27	2.84E-25
	MSTRG.12193.5	186.6036667	90.569	-1.042888101	6.21E-07	6.45E-06
	MSTRG.9372.1	138.0926667	409.9326667	1.569750252	3.01E-09	4.23E-08
	MSTRG.7514.1	127.1483333	634.7096667	2.319584264	1.75E-26	8.46E-25
	MSTRG.1035.1	74.02166667	31.50333333	-1.232443133	6.09E-21	2.16E-19
	MSTRG.5330.1	64.14	29.53666667	-1.118717333	3.24E-18	9.56E-17
	MSTRG.9376.1	62.298	142.0096667	1.188731385	0.000330661	0.001969306
	MSTRG.8578.1	59.252	118.6926667	1.002295045	4.43E-06	3.92E-05
	MSTRG.1407.1	58.93833333	865.177	3.875717144	1.91E-111	7.69E-109
ABZ-resistant expression level (top 10)	MSTRG.11920.3	487.8256667	2694.379	2.465515227	5.63E-27	2.84E-25
	MSTRG.11914.8	18.194	1008.324667	5.792353673	4.68E-74	9.44E-72
	MSTRG.11914.7	14.02066667	870.6436667	5.956455525	9.68E-123	1.17E-119
	MSTRG.1407.1	58.93833333	865.177	3.875717144	1.91E-111	7.69E-109
	MSTRG.11918.1	6.501333333	663.1786667	6.672518163	2.61E-104	7.89E-102
	MSTRG.7514.1	127.1483333	634.7096667	2.319584264	1.75E-26	8.46E-25
	MSTRG.1409.1	54.59966667	430.3133333	2.978423495	2.66E-42	2.30E-40
	MSTRG.9372.1	138.0926667	409.9326667	1.569750252	3.01E-09	4.23E-08

	MSTRG.11914.4	7.252333333	310.4076667	5.419575144	1.82E-116	1.10E-113
	MSTRG.10134.2	51.94933333	269.805	2.376739944	8.90E-26	4.14E-24

Table S4. DElncNRNA information of *trans*-regulation between ABZ-sensitive and ABZ-resistant strains of *Haemonchus contortus*.

**LncRNA ID**	**ABZ-senstive lncRNA FPKM mean**	**ABZ-resistant lncRNA FPKM mean**	**log2(fc)**	**Target mRNA ID**	**ABZ-senstive mRNA FPKM mean**	**ABZ- resistant mRNA FPKM mean**	**log2(fc)**
MSTRG.1010.1	0.259	1.789	2.788	HCON_00110560	9.327	3.93	-1.247
MSTRG.10123.1	9.101	2.114	-2.106	HCON_00110560	9.327	3.93	-1.247
MSTRG.1035.1	74.022	31.503	-1.232	HCON_00110560	9.327	3.93	-1.247
MSTRG.10538.3	0.932	12.234	3.714	HCON_00110560	9.327	3.93	-1.247
MSTRG.10779.1	2.774	38.998	3.813	HCON_00110560	9.327	3.93	-1.247
MSTRG.10904.2	18.963	5.032	-1.914	HCON_00110560	9.327	3.93	-1.247
MSTRG.11077.2	2.726	0.305	-3.158	HCON_00110560	9.327	3.93	-1.247
MSTRG.11438.1	2.009	8.914	2.149	HCON_00110560	9.327	3.93	-1.247
MSTRG.11686.1	25.598	9.678	-1.403	HCON_00110560	9.327	3.93	-1.247
MSTRG.11688.1	48.981	22.7	-1.109	HCON_00110560	9.327	3.93	-1.247
MSTRG.11841.1	12.831	77.801	2.600	HCON_00110560	9.327	3.93	-1.247
MSTRG.11848.1	5.001	1.213	-2.043	HCON_00110560	9.327	3.93	-1.247
MSTRG.11903.1	9.335	109.489	3.551	HCON_00110560	9.327	3.93	-1.247
MSTRG.11914.8	18.194	1008.325	5.792	HCON_00110560	9.327	3.93	-1.247
MSTRG.11918.1	6.501	663.179	6.672	HCON_00110560	9.327	3.93	-1.247
MSTRG.11920.1	0.677	4.052	2.582	HCON_00110560	9.327	3.93	-1.247
MSTRG.12264.9	8.419	3.829	-1.136	HCON_00110560	9.327	3.93	-1.247
MSTRG.13012.1	2.386	15.713	2.719	HCON_00110560	9.327	3.93	-1.247
MSTRG.15545.1	17.97	0.945	-4.249	HCON_00110560	9.327	3.93	-1.247
MSTRG.2293.2	4.081	1.501	-1.443	HCON_00110560	9.327	3.93	-1.247
MSTRG.2387.1	1.586	0.232	-2.771	HCON_00110560	9.327	3.93	-1.247
MSTRG.2710.1	0.893	14.072	3.979	HCON_00110560	9.327	3.93	-1.247
MSTRG.2764.2	3.729	0.871	-2.098	HCON_00110560	9.327	3.93	-1.247

MSTRG.3269.2	1.083	0.108	-3.330	HCON_00110560	9.327	3.93	-1.247
MSTRG.3343.1	0.489	29.743	5.928	HCON_00110560	9.327	3.93	-1.247
MSTRG.3345.1	1.299	21.968	4.080	HCON_00110560	9.327	3.93	-1.247
MSTRG.3492.1	12.639	4.494	-1.492	HCON_00110560	9.327	3.93	-1.247
MSTRG.5313.1	0.933	15.559	4.059	HCON_00110560	9.327	3.93	-1.247
MSTRG.5330.1	64.14	29.537	-1.119	HCON_00110560	9.327	3.93	-1.247
MSTRG.5476.1	7.359	14.831	1.011	HCON_00110560	9.327	3.93	-1.247
MSTRG.5669.1	2.856	1.32	-1.113	HCON_00110560	9.327	3.93	-1.247
MSTRG.5755.2	2.347	22.381	3.253	HCON_00110560	9.327	3.93	-1.247
MSTRG.5895.1	55.852	19.865	-1.491	HCON_00110560	9.327	3.93	-1.247
MSTRG.5968.1	0.728	9.951	3.772	HCON_00110560	9.327	3.93	-1.247
MSTRG.7238.4	18.226	132.358	2.860	HCON_00110560	9.327	3.93	-1.247
MSTRG.7300.1	2.289	0.186	-3.619	HCON_00110560	9.327	3.93	-1.247
MSTRG.7322.1	2.933	30.135	3.361	HCON_00110560	9.327	3.93	-1.247
MSTRG.7325.1	2.183	27.085	3.633	HCON_00110560	9.327	3.93	-1.247
MSTRG.7514.1	127.148	634.71	2.320	HCON_00110560	9.327	3.93	-1.247
MSTRG.7719.1	2.168	0	-11.082	HCON_00110560	9.327	3.93	-1.247
MSTRG.8336.1	2.285	0.475	-2.267	HCON_00110560	9.327	3.93	-1.247
MSTRG.8470.1	46.32	13.071	-1.825	HCON_00110560	9.327	3.93	-1.247
MSTRG.8581.1	1.758	107.18	5.930	HCON_00110560	9.327	3.93	-1.247
MSTRG.8654.1	2.715	0.983	-1.466	HCON_00110560	9.327	3.93	-1.247
MSTRG.8672.2	1.59	0	-10.635	HCON_00110560	9.327	3.93	-1.247
MSTRG.871.1	1.819	0	-10.829	HCON_00110560	9.327	3.93	-1.247
MSTRG.8984.1	0.463	5.452	3.557	HCON_00110560	9.327	3.93	-1.247
MSTRG.9827.1	3.187	0	-11.638	HCON_00110560	9.327	3.93	-1.247
MSTRG.1010.1	0.259	1.789	2.788	HCON_00110560	9.327	3.93	-1.247
MSTRG.10123.1	9.101	2.114	-2.106	HCON_00110560	9.327	3.93	-1.247
